# Prognosis of Tumor Microenvironment in Luminal B-Type Breast Cancer

**DOI:** 10.1155/2022/5621441

**Published:** 2022-02-10

**Authors:** Ji Lv, Jia Ren, Jie Zheng, Fuliang Zhang, Meng Han

**Affiliations:** Department of Surgery, The First Hospital of Qinhuangdao, Qinhuangdao City, Hebei Province, China

## Abstract

**Objective:**

Tumor microenvironment as an important element of malignancy could help predict cancer prognosis and therapeutic response; thus, a prognostic landscape map of the tumor microenvironment in luminal B breast cancers should be developed.

**Methods:**

The GEO and TCGA databases were employed to retrieve clinical follow-up data and expression profiles of luminal B breast cancer. CIBERSORT was applied to assess the infiltration of the tumor microenvironment of 209 patients and to construct tumor microenvironment-based subtypes of luminal B breast cancer. We also conducted Cox multivariate regression analysis to select features that could be used to develop a microenvironment signature for cancer. Samples were categorized as having low and high TME scores according to the median TME score. The correlations of prognosis and TME score, expression levels of immune factors and genomic variation, and clinical features were further investigated.

**Results:**

We found that high TME scores were correlated with poor prognosis. The current findings showed that the expressions of multiple immune-related genes, including CXCL9, CXCL10, GZMB, and PDCD1LG2, were upregulated in cancer with high TME scores. The high-risk group showed lower TP53 gene mutation frequency as opposed to that of the low-risk group. For the purpose of developing a TME scoring system, the TME infiltration levels of 209 patients with luminal B breast cancer from TCGA were comprehensively analyzed.

**Conclusions:**

Our analysis revealed that the TME score was an indicator of patients' response to immune checkpoint modulators and an effective prognostic biomarker. TME scoring improves current immunotherapy on luminal B breast cancer.

## 1. Introduction

Statistics on breast cancer showed that in 2018, over 2.1 million cases of breast cancer were recorded, resulting in about 630,000 breast cancer deaths [1]. Recent advancements in genomics have greatly improved our comprehension of the molecular basis that underlies breast cancer. Based on the intrinsic gene expression of breast cancer, the concept of molecular typing was first proposed in 2000 [2, 3]. Four subtypes of cancer include HER-2 overexpression type, basal type, and luminal types A and B [4, 5].Endocrine therapy is often implemented to treat most luminal B-like breast cancer, the recurrence of which is often fatal. The improvement of the treatment outcomes also requires a better understanding of the development of new prognostic markers.

Tumor microenvironments (TMEs) are created at various stages of tumor growth. TMEs have been found to support tumorigenesis, as they could influence immune cell activation, which in turn promote tumor growth and progression [6]. Multiple studies showed that TME is involved in the progression of cancer and patients' responsiveness to therapy [7, 8]. Previous cancer studies detected differences in components of resident cell types in TME, such as mesenchymal stem cells, tumor-associated macrophages, cytotoxic T cells, dendritic cells (DCs), helper T cells, and associated inflammatory pathways [9–12]. Assessment of patients' TME at diagnosis may provide insight into immune responses to cancer and cancer response to chemotherapy [13]. Alterations in the proportions of infiltrating macrophages, CD8+ T cells, fibroblasts, and CD4+ T cells, in TME are associated with clinical progression of cancer and may be prognostically significant in various cancers [12, 14–16]. Multiple algorithms developed for estimating the abundance of immune cells along with other cell types in TME [17–19] were previously applied to explore the association between TME infiltration and cancer progression [20, 21]. Nevertheless, no comprehensive investigations have been performed on the TME landscape infiltration in luminal B breast cancers.

In this research, we analyzed annotations of gene expression profiles derived in clinical luminal B breast cancer samples using the CIBERSORT algorithm. In this way, we identified 22 immune cell types and cancer-associated fibroblast proportion. After evaluating 209 patients with luminal B breast cancer, systematic characterization of the TME phenotype, clinical pathologic features, and the luminal B samples related to breast cancer genome was performed. Here, we described a strategy for quantifying the infiltration pattern of the TME score, which showed prognostic significance and had the potential to predict breast cancer response to immune checkpoint inhibitors.

## 2. Materials and Methods

### 2.1. Data Acquisition and Processing

The expression profile data of TCGA Affymetrix HT Human Genome U133a microarray platform and accompanying prognostic information were downloaded from the GDC API database on 14^th^ August 2019. We excluded samples that did not contain clinical information or with follow-up durations that were less than 30 days. A sum of 209 luminal B breast cancer samples fulfilled the eligibility requirements for the final research and were used as a training set. The chip datasets (GSE21653 [22, 23] and GSE1456 [24, 25]) were downloaded from the gene expression omnibus (GEO) [26]. The datasets had clinical follow-up and expression data of 65 luminal B breast cancer samples in total from Affymetrix Human Genome U95 Version 2 array (http://www.affymetrix.com/support/technical/byproduct.affx?product=hgu95), Affymetrix Human Genome U133A Array (http://www.affymetrix.com/support/technical/byproduct.affx?product=hgu133-20), and Affymetrix Human Genome U133 Plus 2.0 Array (Affymetrix Human Genome U133 Plus 2.0 Array) (the duration for follow-up was over 30 days). Next, transcriptome quantification of the unified pipeline was performed on each sample, and the overall abundance factor was used for correction. All the samples analyzed in this study were collected before standard treatment (Table 1); we analyzed according to the workflow of Figure S1A.

The probe data were mapped into the GeneSymbol with the R package hgu133plus2.db. In the case where multiple probes were mapped into one gene, the median of the gene expression was taken. The probes matching to multiple genes were excluded.

### 2.2. TME Score for Infiltrating Cells

CIBERSORT, which is a commonly utilized deconvolution algorithm, incorporates reference gene expression levels considered to minimally represent different cell types and uses the signatures to infer various cell types in tumor samples. Combined with support vector regression, CIBERSORT can identify 22 human immune cells, including NK cells, macrophages, T cells, myeloid subset cells, B cells, and DCs with high sensitivity and specificity. As a reference, the LM22 gene signature was applied to quantify immune cells in the cancer samples in this study; then, to develop an LM22 signature, CIBERSORT [17] (http://cibersort.stanford.edu/) was applied to detect 22 different types of immune cells in TCGA and GSE13041 datasets through uploading the gene expression into the algorithm. 1000 times of permutation were then performed and scored to identify the 22 immune cell types.

### 2.3. Consensus Clustering-Derived Molecular Subtypes Correlated with TME-Infiltrating Cells

Consensus clustering was conducted utilizing the Consensus ClusterPlus software in R to identify subgroups of patients with luminal B-type breast cancer based on the presence of TME-infiltrating cells [27]. As previously described [28], to ensure the stability of the findings, the optimal number *k* of clusters ranged between 2 and 10, while repeating the procedure 1000 times. The R software program was used to visualize the results of this investigation.

### 2.4. Differentially Expressed Genes (DEGs) Correlated with the TME Phenotype

It was necessary to employ a linear model to evaluate gene expression differences across TME phenotypic subgroups in order to discover genes correlated with TME cell infiltration patterns. In particular, the R software tool DESeq2 was utilized [29] to compute differential gene expression. We chose an FDR of less than 0.05. Fold change was not limited in order to allow the inclusion of more potential genes.

### 2.5. TME Phenotype-Related Differential Gene Reclustering

Nonnegative matrix factorization (NMF) is a widely utilized unsupervised clustering approach that has been extensively employed for the identification of tumor subgroups based on genomic data [30, 31]. In this regard, NMF was utilized to recluster samples based on their TME phenotypic and differential gene expression profiles in order to assess the association between phenotype and TME phenotype-related differential gene expression. Subsequently, the clinical characteristics of the reclustered samples were examined. After 50 repetitions, NMF chose the standard “brunet.” NMF, an R package, was used to compute an averaged profile width of the common member matrix for each of the clusters with a *k* value ranging from 2 to 10 [32]. Each subclass had a minimum membership of ten.

### 2.6. Establishing TME Gene Signatures and Reducing the Dimension

To develop a robust TME gene signature, we employed the random forest algorithm to evaluate the significance of differentially expressed genes (DEGs). For univariate survival analysis, the copth function of survival in the R package was employed. A cutoff value of 0.05 was set for the selection of DEGs. We used the random forest R program to input genes with considerable prognostic value into the random forest feature selection process. Each segment was assigned a mtry of 1–235 as well as a ntree of 500. The value of mtry with the least error value was chosen as the ideal factor for the random forest method, and an ntree of 100 was chosen on the basis of the error rate of the random forest. Lastly, the DEGs were ordered according to their significance. DEGs with >95% cumulative importance were selected as candidate feature genes. The genes were then grouped into 5 categories according to *K*-means [33]. The 5 categories were then analyzed utilizing the R Psych package. After 100 repetitions, the signature score was derived from the first principal component. For gene type *j*, the following formula was used to calculate scores of the signature:

(1) $$\begin{array}{c} {\text{S}}_{j}=\overset{n_{j}}{\underset{i=1}{\sum }}\text{P}\text{c}1_{i}*{\text{Exp}}_{i}, \end{array}$$

where *j* denotes the *j*^th^ class of the 5 different kinds of genes,  *n*_*j*_ denotes the count of genes of the *j*^th^ gene, Pc1_*i*_ denotes the coefficient of the first principal component of the *i*^th^ gene of the *j*^th^ gene, and Exp_*i*_ denotes the initial level of the *i*^th^ gene expression of the *j*-type gene.

Signatures G1, G2, and G3 were subjected to a PCA in R utilizing the psych function. Principal component (PC) scores were determined for each gene signature after 100 repetitions in order to acquire the optimal number of PCs. The ultimate score was determined as the PC1 values for G1, G2, and G3. Subsequently, Cox multivariate regression was performed to compute the risk score coefficient for the gene signature in each of the three groups (G1, G2, and G3). TME scores for each sample were computed using the following equation:

(2) $$\begin{array}{c} \text{TME score}=\sum \text{P}\text{C}1*\propto . \end{array}$$

In this equation, each gene signature coefficient for multivariate regression is represented by ∝, while the PCI score of each signature is represented by PCI.

### 2.7. Correlation between Clinical Characteristics and TME Score

To clarify the relationship between TME score and clinical phenotype, we classified the samples into two groups according to their median TME score and compared the prognostic differences between low and high TME scores. The association between a lower or higher TME score and the variables of age and gender was investigated.

### 2.8. Correlation between the TME Score and the Expression of Immune-Related Genes

Three categories of immune-related genes were chosen in order to examine the association between TME score and these genes: (1) immune activation genes, including CD8A, IFNG, GZMA, TBX2, PRF1, CXCL9, TNF, GZMB, and CXCL10 [34]; (2) immune checkpoint genes, including HAVCR2, LAG3, PDCD1LG2, CD274, IDO1, CTLA4, and PDCD1 [35]; and (3) TGF/EMT pathway genes, including TWIST1, COL4A1, SMAD9, ZEB1, TGFBR2, CLDN3, ACTA2, and VIM [36]. In order to further investigate the differences in the expression of the three gene categories between low and high TME scores, their expression profiles were investigated.

### 2.9. Relationship between TME Score and Tumor Genomic Variation

In order to compare genomic differences across samples with low and high TME scores, we obtained SNP data from TCGA and deleted silent mutations and introns before analyzing the results. Fisher's exact test was then performed to assess mutation differences between the two sample classes by setting the selection threshold at *p* < 0.05.

### 2.10. Statistical Analysis

Unless otherwise stated, the Shapiro-Wilk normality test [37] was utilized to determine the normality of the variable. The unpaired Student *t*-test was utilized to evaluate whether the normally distributed variables had significance in the two groups. The Mann–Whitney *U* test was employed to examine variables exhibiting nonnormal distributions. To compare parametric and nonparametric variables, one-way ANOVA and Kruskal-Wallis test were employed [38]. The correlation coefficients were obtained utilizing the distance and Spearman correlation analyses. The contingency table analysis was carried out utilizing a two-sided Fisher exact test. The *p* value was converted to the FDR by applying the Benjamini-Hochberg technique. For each dataset, Kaplan-Meier survival analysis was conducted on subgroups to determine their survival. The log-rank test was employed to examine the significance of the data. *p* value < 0.05 was defined as statistically significant. In all cases, unless otherwise stated, statistical analyses were performed in R (version: 3.4.3) with default configuration.

## 3. Results

### 3.1. TME Landscape regarding the Luminal B Breast Cancer

**Figure 1:**
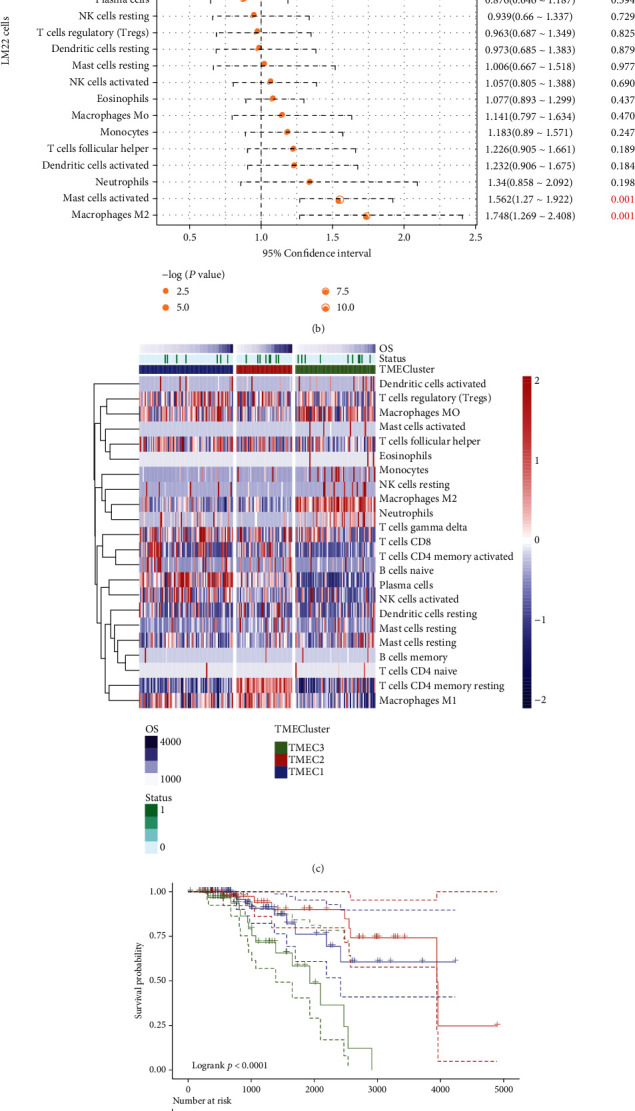
(a) Relationships of 22 different immune cell types in TME. The color and size of the dot indicate correlation, where white color denotes insignificant, red color denotes positive correlation, and blue color denotes negative correlation. (b) Forest map showing the 22 distinct types of immune cells. (c) Heat map showing the 22 distinct types of immune cell scores in TME, with the lower score being indicated by the basket whereas the higher score is indicated by the redder. (d) This KM curve shows the three different forms of TMEC and their corresponding OS prognosis of patients. (e) The box plots depict the distribution of 22 immune cell scores across the three TMEC categories; red^∗^ denotes obvious differences.

The functions of diverse immune cell types in luminal B breast cancer TME were examined using CIBERSORT, which could examine the association of 22 distinct immune cell scores from 209 tumor tissues. A considerable positive association between the four types, for instance, immune activation cells, was observed, indicating a particular mode of communication of immune cells (Figure 1(a)). The relationship between breast cancer prognosis and 22 immune cell scores was analyzed by univariate Cox regression. The results showed that the scores of M2 macrophages and activated mast cells were strongly associated with an unfavorable prognosis (log-rank *p* < 0.05, HR > 1). In contrast, the scores of activated CD4 memory T cells and M1 macrophages were correlated with a favorable prognosis (HR < 1, log-rank *p* < 0.05, Figure 1(b)). Firstly, the scores for the 6 types of immune cells showing a strong correlation with prognosis were recruited into ConsensusClusterPlus to evaluate the optimum clustering factor *k* ranging between 2 and 10. This operation was repeated for a total of 1000 times. According to the delta area and CDF value, the optimal clustering factor *k* was 3 (Figure S1B-D). TME scores were then categorized into 3 classes (Supplementary Table 1), TMEC1, TMEC2, and TMEC3. The results demonstrated that immune cells, such as naïve B cells and regulatory T cell (Treg) scores, were noticeably higher in TMEC1 and that M1 macrophages M1 and CD4 T cells scores were greatly higher in TMEC2. NK cells and M2 macrophages showed elevated scores in TMEC3 (Figure 1(c)). The results from the analysis on overall survival (OS) revealed significant differences in OS among three TMECs (log-rank *p* < 0.0001). Specifically, TMEC2 indicated the optimal prognosis, TMEC3 was indicative of the poorest prognosis, while TMEC1 was intermediate between the two classes (Figure 1(d)). Statistically significant differences between the prognostic significance of TMEC1, TMEC2, and TMEC3 were observed, but no obvious differences were found between TMEC2 and TMEC1 (Figure S2A–C). In addition, we obtained mRNA-based molecular subtypes from previous studies of Berger et al. [39]. By comparing the intersection of TMEC1-3 and mRNA subtypes, we can observe that the C7 subgroup of Berger et al. mainly comes from TMEC3, and the C8 subgroup mainly comes from TMEC1. In addition, there are significant differences in the distribution of TMEC subtypes in C1 and C2 subgroups of Berger et al. (Figure S2D). 12 out of 22 immune cell type scores exhibited statistically significant differences in prognosis (Figure 1(e)). Taken together, these data showed that the TME scores may closely mirror the development of cancer.

### 3.2. Functional Analysis and DEGs between TMEC

**Figure 2:**
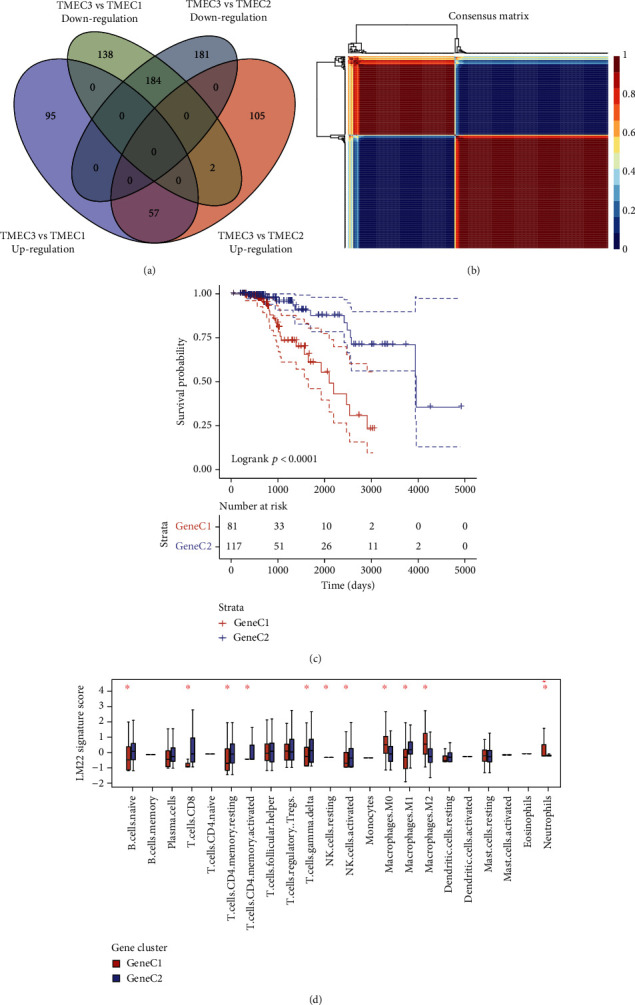
(a) Venn diagrams illustrating upmodulated DEGs in response to TMEC1, TMEC2, and TMEC3. (b) Consistency matrix for the NMF algorithm as depicted by a heat map. (c) OS prognostic KM curve for Gene C1 and Gene C2. (d) The box plot shows the scores for 22 immune cells in the Gene C1 and Gene C2 samples.

Differences in gene expression of various TMECs were analyzed with differential TMEC gene expression in TCGA data. DEGs between TMEC1, TMEC3, and TMEC2 were calculated using the DESeq2 tool. Current analyses detected 241 common DEGs between TMEC1/TMEC3 and TMEC2/TMEC3 (Figure 2(a)). Next, from the 241 DEGs, scorings 0 in 50% of the samples were filtered; here, a total of 235 genes were recruited into further analyses. NMF, which was then used for reclassification analysis of the TCGA samples, generated 2 stable classes, namely, Gene C1 and Gene C2 (Figure 2(b)). Survival analysis revealed significant differences in the survival between Gene C1 and Gene C2 (Figure 2(c)). The analysis of Gene C1 and Gene C2 distribution across the 22 immune cell types demonstrated that their distribution in various TMEs was significantly different. For example, Gene C1, which exhibited the poorest prognosis, showed a substantially elevated score in macrophages M2, macrophages M1, and macrophages M0, when compared with Gene C2 (Figure 2(d)).

### 3.3. A TME Signature Development

**Figure 3:**
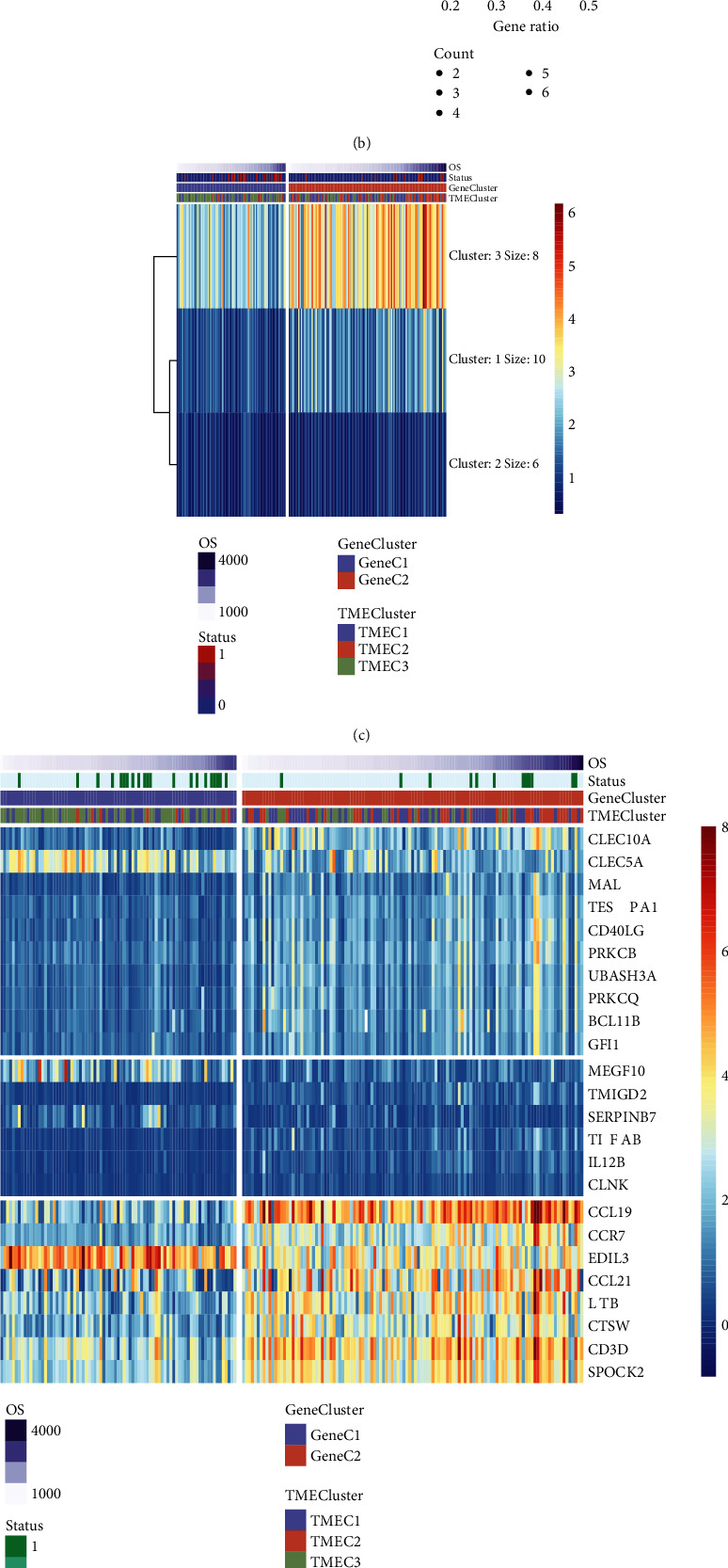
(a) 24 genes were enriched in GO terms. (b) 24 genes were enriched in KEGG. (c) Results of the *K*-means clustering of 24 genes. (d) Gene expression heat maps for 24 genes. (e) TME score for Gene C1 and Gene C2. (f) Gene C1 and Gene C2 TME score distributions. (g) OS prognosis in the risk-h and risk-l groups illustrated by KM curve.

The 235 DEGs shared by the 3 TMECs were screened; their importance was assessed using the random forest package on R. ntree = 100 was selected based on the random forest (Figure S3A), and 24 candidate genes were detected from DEGs with a cumulative significance greater than 95% (Figure S3B-C). GO term and KEGG analysis revealed that the 24 genes were enriched to primary immunodeficiency, NF−kappa B signaling, T cell activation, and some immune-related pathways (Figures 3(a) and 3(b)). Next, the genes were grouped into 3 signature clusters (Figure 3(c)), namely, signature G1 (10 genes), G2 (6 genes), and G3 (8 genes), by the *K*-means algorithm (Figure 3(c)). Signature cluster G1 was found in the intermediate group, G2 was found in the group with low expression, while G3 was found in the high expression group (Figure 3(d)).

The TME score of samples in the training set was counted by developing a TME score system. A comparison of the TME scores of Gene C revealed a significantly higher proportion of worse prognosis of Gene C1 than the optimal prognosis of Gene C2 (Figures 3(e) and 3(f)). Next, the samples were classified into two categories (risk-h and risk-l) according to the median TME score (0.3657). The analysis showed a considerably different prognosis between the risk-l group and risk-h group (HR = 2.928, log-rank *p* = 0.0024) (Figure 3(g)).

### 3.4. Correlation among TME Score Clinical Features and Immunological Gene Expression

**Figure 4:**
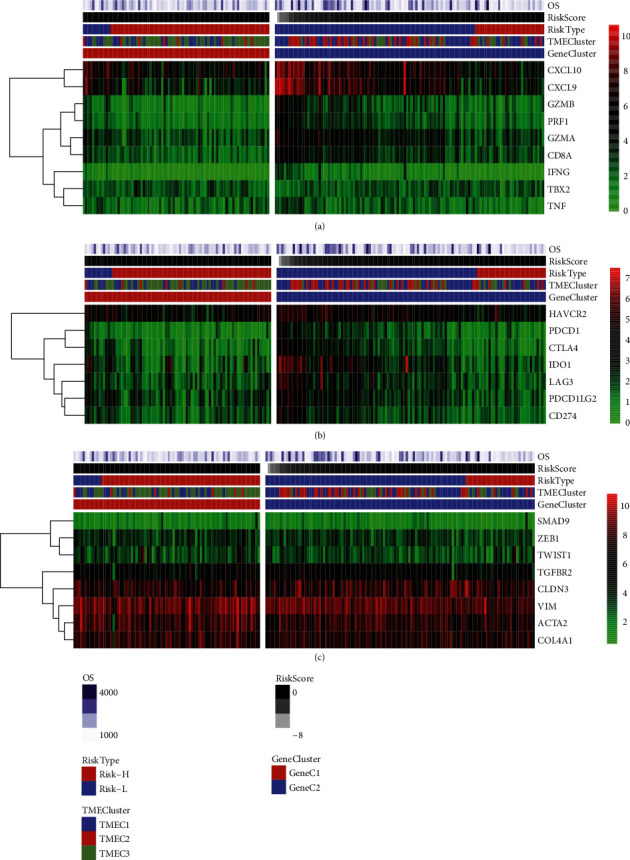
(a) Expression of immune activation genes in TCGA samples as shown graphically in a heat map. (b) Expression of immune checkpoint genes in TCGA samples as shown graphically in a heat map. (c) The heat map depicts the expression of TGF pathway genes in TCGA samples.

The evaluation of the correlation between clinical features and TME scores did not reveal any significant correlation (Figure S4). To examine the relationship between immunological status and different TMEs, the correlation of TMEC and expression of immune activation genes (TBX2, CXCL10, GZMB, IFNG, GZMA, CD8A, PRF1, TNF, and CXCL9) of Gene C and TME scores was analyzed. Here, the results demonstrated various distinct patterns of expression in TME scores, Gene C, and TMECs. Specifically, the expression of CXCL9 and CXCL10 in the high-risk group, which was correlated with an unfavorable prognosis, was noticeably elevated in the group with low-risk (Figure 4(a)). Analysis of the correlation between the expression of immune checkpoint genes (HAVCR2, PDCD1LG2, CD274, LAG3, PDCD1, IDO1, and CTLA4) and TME score, TMEC, Gene C revealed that although the genes were not high-expressed, genes of different Gene Cs, TME scores, and TMECs exhibited different expression trends. IDO1 and HAVCR2 expressions in the risk-h were noticeably elevated as opposed to those in the risk-l group (Figure 4(b)). The analysis of expression of TGF/EMT pathway factors (TWIST1, ACTA2, ZEB1, TGFBR2, CLDN3, COL4A1, SMAD9, and VIM) in TME score, TMEC, and Gene C showed limited differences in gene expression in Gene C, TME score, and TMEC, with only TGFBR2 showing a noticeably higher level in the risk-h group when compared to the risk-l group (Figure 4(c)). Likewise, the same trend was identified in three distinct sets of TME samples (Figure S5, S6). The above observations indicated that the TME score was correlated with the level of immune genes.

### 3.5. TME Features of a Tumor Genome

**Figure 5:**
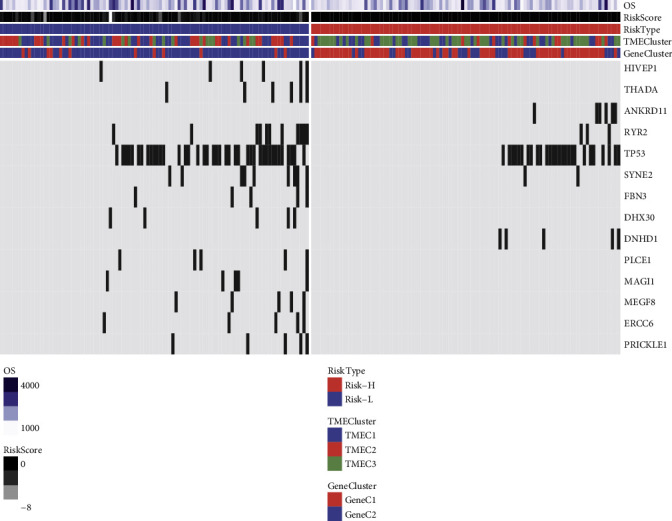
The correlation between TME and the features of genetic mutations. Samples are shown on the horizontal axis, while genes are represented on the vertical axis, mutations are represented by the black rectangle, while the absence of mutations is represented by the gray rectangle.

According to the patients' TME scores, they were categorized into risk-l and risk-h groups. After investigating the correlation between genomic variation and TME score, TME score-associated genes were identified. After removing intron and silent mutations, a Fisher test was performed to analyze genes showing considerable differences in mutation frequency between the two groups. Here, 14 genes with prominent mutation frequencies were detected (Figure 5), and TP53 mutation frequency was elevated in risk-l as opposed to that in risk-h; in luminal B breast cancer, these genes were closely associated with TME.

### 3.6. Comparison of Risk Models with Other Models

**Figure 6:**
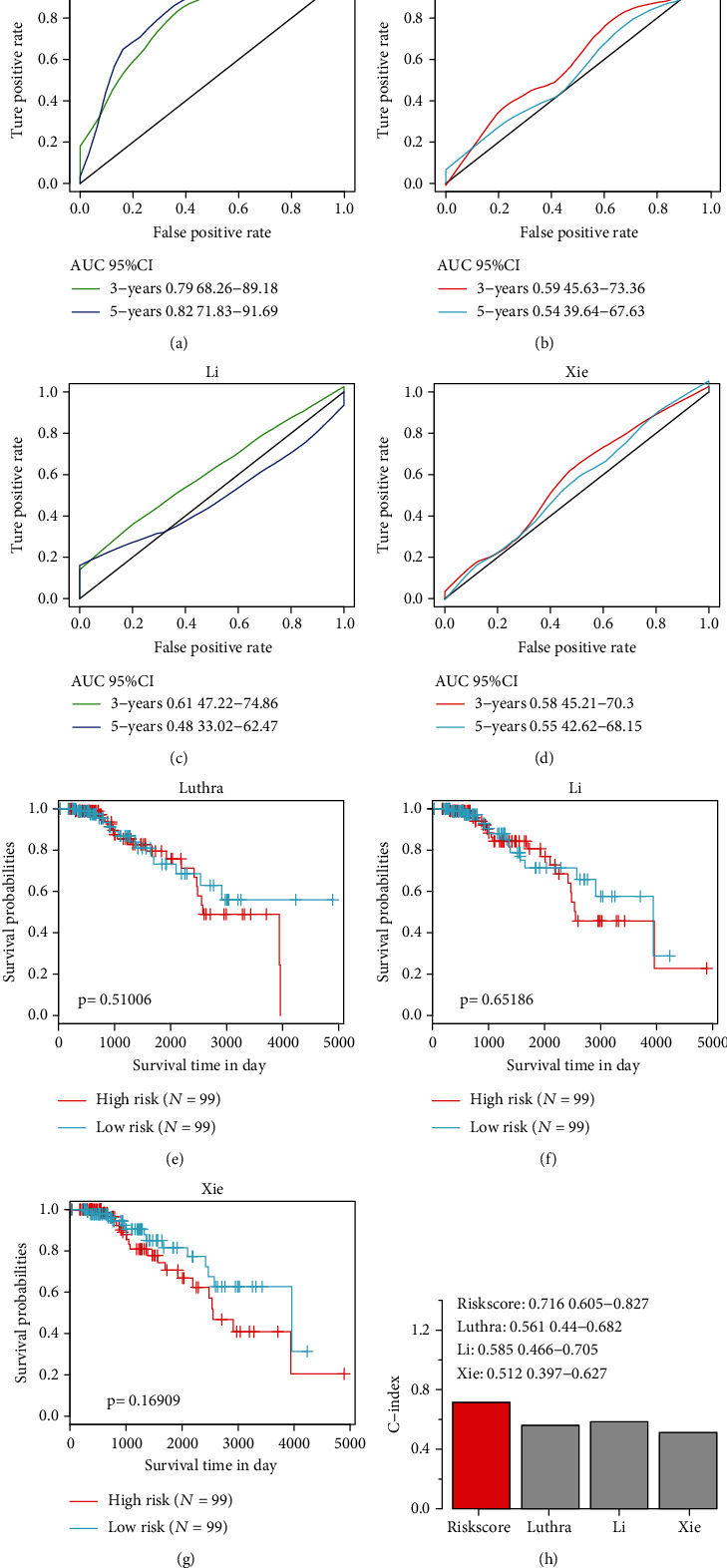
(a–d) ROC curves for four different models. (e–g) The survival curves for three different models. (h) *C*-index of four models.

We selected 3 risk models associated with prognosis, namely, a 4-gene signature by Luthra et al. [40], a 4-gene signature by Li et al. [41], and a 4-gene signature by Xie et al. [42]. The three previously developed gene signature systems were compared with our TME score model. To promote the comparability of the models, risk scores for the luminal B breast cancer samples acquired from TCGA were calculated by the same method; moreover, ROC of the model was assessed by median risk score. Next, the samples were categorized into risk-l and risk-h groups and we calculated the OS differences. The analysis showed a 5-year AUC of TME score of 0.82, with a ROC of the other three models, and only the model developed by Li et al. showed a three-year AUC higher than 0.61 (Figures 6(a)–6(d)). The risk-l and risk-h groups did not exhibit considerable differences in the prognostic KM curves for the three models (Figures 6(e)–6(g)). To analyze the ability of these models in recognizing samples of luminal B breast cancer and to estimate the concordance index (*C*-index) of our TME score model as well as the 3 models, we utilized the RMS function in R. As illustrated in Figure 6(h), our TME score model appeared to have the greatest *C*-index, which indicated that it performed much better as opposed to the other three models.

## 4. Discussion

Cancer is a leading cause of mortality worldwide. Previous research has primarily focused on tumor cells. Paget [43] put forward the “seed and soil” theory, bringing the concept of TME into cancer research in 1989. Progress in cancer research has now established that tumors are complete tissues with unique homeostasis [44]. Over time, the focus of cancer study has progressively changed from cancer cells to tumors and their microenvironment.

In the tumor microenvironment, immune responses and tumor-infiltrating immune cells have been paid great attention for their potential of serving as therapeutic targets. Recently, immune checkpoint inhibitors have exhibited antitumor properties against advanced cancers, including in breast cancer [45]. We analyzed the TME landscape in luminal B breast cancer and discovered that various immune cell types were correlated with an unfavorable prognosis of patients with luminal B breast cancer and could be used to molecularly stratify luminal B breast cancers. In addition, as breast cancer is a nonimmune disease, few immunotherapies are applied to breast cancer. In metastatic breast cancer, tumor vaccine has shown good antitumor activity, but objective remission rate is low. Recent studies have shown that PAM50-typed lumb rather than luma subtype has the characteristics of acquired immune response activation. The tumors of lumb subtype show immune microcyclic activation, which may be a potential benefit group of immunotherapy [46]. In this study, we identified three molecular subtypes of lumb, which had significantly different immune infiltrations. These differences may indicate different degrees of benefit from immunotherapy. Moreover, this also indicated that the immune features of cancers differed across various stages of breast cancer development.

Research reports have indicated that in the TME, T cells are regulated by both inhibitory and stimulating signals. The immune checkpoint is a molecular “switch” for inhibition. Previous study [47] analyzed 1861 advanced melanoma patients and showed that anti-CTLA4 antibody (ipilimumab) could greatly prolong patients' long-term survival. Recently, anti-PD1 antibody (nivolumab) has been found to be ineffective against metastatic renal cell carcinoma, advanced squamous cell carcinoma, non-small-cell lung cancer, melanoma, or luminal-like breast cancer [48, 49]. Therefore, there is a need for developing novel biomarkers predictive of whether cancer patients could benefit from checkpoint immunotherapy. Research indicated that TME is critical in checkpoint inhibitor immunotherapy [50]. PDL1 blockade could be attenuated by modulating the small cell lung cancer immune microenvironment [51]. Ubago et al. found that >50% of tumor-infiltrating lymphocytes in HER2 breast tumors express PDL1, while about a third express PD1 [52]. The memory of tumor immunotherapy and immune microenvironment could be reshaped and enhanced by multifunctional nanoregulator [53]. In this study, we thoroughly examined the landscape interplayed between TME infiltration cells and luminal B breast cancer clinical features. Through using various computational tools, we designed an infiltrating pattern of the TME-TME score.

The current findings discovered that the TME score was a prognostic indicator of luminal type B breast cancer, with an elevated TME score relating to an unfavorable prognosis of breast cancer. After detecting the liver tissues of early-staged HCC patients, a previous study demonstrated a higher risk of HCC is correlated with the pattern of immune-related gene expression [54]. A novel immunotyping and immune gene signature was established for luminal B-like breast cancer according to the immune gene expression profiles [55]. A study reported the STAT1/NK axis activation in TME could predict immune checkpoint blockade response of patients, suggesting that a biomarker-driven treatment strategy can predict whether patients might benefit from sensitizing therapeutics prior to immune checkpoint blockade [56]. Here, we found that elevated levels of immune activation genes (e.g., CXCL10, GZMB, and CXCL9) and immune checkpoint genes (e.g., PDCD1 and CTLA4) were correlated with high TME scores. The results indicated that TME scores may predict those patients who can benefit from immune checkpoint blocking therapy. TP53 is frequently mutated in breast cancer and is important for the treatment and prognosis of this disease [57]. Zhu et al. clustered luminal breast tumor subtypes characterized by an elevation of TP53 somatic mutations [58]. TP53 mutation frequencies in samples with a high TME score were greatly lower than in those with low TME scores. The results opened the new door for studying mechanisms of TME formation as well as the role of each mutation in immunity and immunotherapy of luminal B breast cancer.

This research has identified potential immune gene biomarkers for luminal type B breast cancer through bioinformatics; further validation is needed to more accurately specify the threshold values using a prospective cohort of immunotherapies. Secondly, considering the heterogeneity of tumors, analysis of the edge of the core and evaluation of the immune cell infiltration are also necessary because patients with high TME scores may not all benefit from immunotherapy. Finally, our results obtained from computational biology require experiments for validation.

## 5. Conclusion

This study examined TME infiltration patterns from a sum of 209 patients with luminal B breast cancer and generated a pipeline for elucidating patterns of TME infiltration. We discovered that the TME score was an excellent prognostic predictor of breast cancer patients' response to immune checkpoint inhibitors.

## Figures and Tables

**Table 1:** Clinical information of the three sets of datasets after pretreatment.

Characteristics		TCGA datasets	GSE1456 (*n* = 23)	GSE21653 (*n* = 42)
Age (years)	≤60	109	—	28
	>60	89	—	14
Survival status	Living	165	16	23
	Dead	33	7	19
Pathologic_T	T1	37	—	—
	T2	127	—	—
	T3	24	—	—
	T4	10	—	—
Pathologic_N	N0	77	—	—
	N1	74	—	—
	N2	33	—	—
	N3	10	—	—
Pathologic_M	M0	168	—	—
	M1/MX	28	—	—
Tumor stage	Stage I	23	—	41
	Stage II	112	—	31
	Stage III	57	—	19
	Stage IV	4	—	3
Pathology	IDC	121	—	34
	ILC	5	—	2
	MIX	24	—	3
	Others	25	—	1

## Data Availability

The data used to support this research were included within this manuscript.

## Supplementary Materials

Supplementary 1 Figure S1: A: work flow chart. B: consensus matrix for TME-infiltrating cell classification with the corresponding heat map. The colour-coded heat map corresponding to the consensus matrix for *k* = 3 obtained by applying consensus clustering. The colour gradients were from 0 to 1, representing the degree of consensus, with white corresponding to 0 and dark blue to 1. C: the cumulative distribution function (CDF) curves in consensus cluster analysis. CDF curves of consensus scores by different subtype numbers (*k* = 2, 3, 4, 5, 6, 7, 8, 9, 10) were represented. D: delta area curve of consensus clustering, indicating the relative change in area under the cumulative distribution function (CDF) curve for each category number *k* compared with *k*–1. The horizontal axis represents the category number *k*, and the vertical axis represents the relative change in area under CDF curve.

Supplementary 2 Figure S2: A: prognosis KM curve of TMC1 vs. TMEC2. B: prognosis KM curve of TMC2 vs. TMEC3. C: prognosis KM curve of TMC1 vs. TMEC3. D: the molecular subtypes of TCGA samples were compared with the mRNA subtypes of Berger et al.

Supplementary 3 Figure S3: A: the distribution of random forest error rate varying with tree parameters. B: multidimensional scaling (MDS) plot for GeneCluster (Gene C1 and Gene C2) data. C: random forest importance for DEGs with mean decrease Gini index (blue) and mean decrease accuracy (red).

Supplementary 4 Figure S4: the relationship between TME scores and clinical characteristics found no significant difference. A: the relationship between TME scores and T stage. B: the relationship between TME scores and N stage. C: the relationship between TME scores and M stage. D: the relationship between TME scores and stage. E: the relationship between TME scores and age.

Supplementary 5 Figure S5: A: the expressions of immune activation genes (CXCL10, CXCL9, GZMA, GZMB, PRF1, IFNG, TBX2, TNF, and CD8A) in risk-l and risk-h samples. B: the expressions of immune checkpoint genes (PDCD1, CTLA4, LAG3, IDO1, CD274, PDCD1LG2, and HAVCR2) in risk-l and risk-h samples. C: the expressions of TGF/EMT pathway genes VIM, ACTA2, COL4A1, TGFBR2, ZEB1, CLDN3, SMAD9, and TWIST1 in risk-l and risk-h samples.

Supplementary 6 Figure S6: A: the expressions of immune activation genes (CXCL10, CXCL9, GZMA, GZMB, PRF1, IFNG, TBX2, TNF, and CD8A) in TMEC. B: the expressions of immune checkpoint genes (PDCD1, CTLA4, LAG3, IDO1, CD274, PDCD1LG2, and HAVCR2) in TMEC. C: the expressions of TGF/EMT pathway genes VIM, ACTA2, COL4A1, TGFBR2, ZEB1, CLDN3, SMAD9, and TWIST1 in TMEC.

Supplementary 7 Supplementary Table 1: molecular subtypes of TCGA samples.
