# Supplementary figures and images for: Prognosis of Tumor Microenvironment in Luminal B-Type Breast Cancer
Source: Dis Markers. 2022 Feb 10;2022:5621441. doi: 10.1155/2022/5621441 (PMC8886761; doi:10.1155/2022/5621441)

A

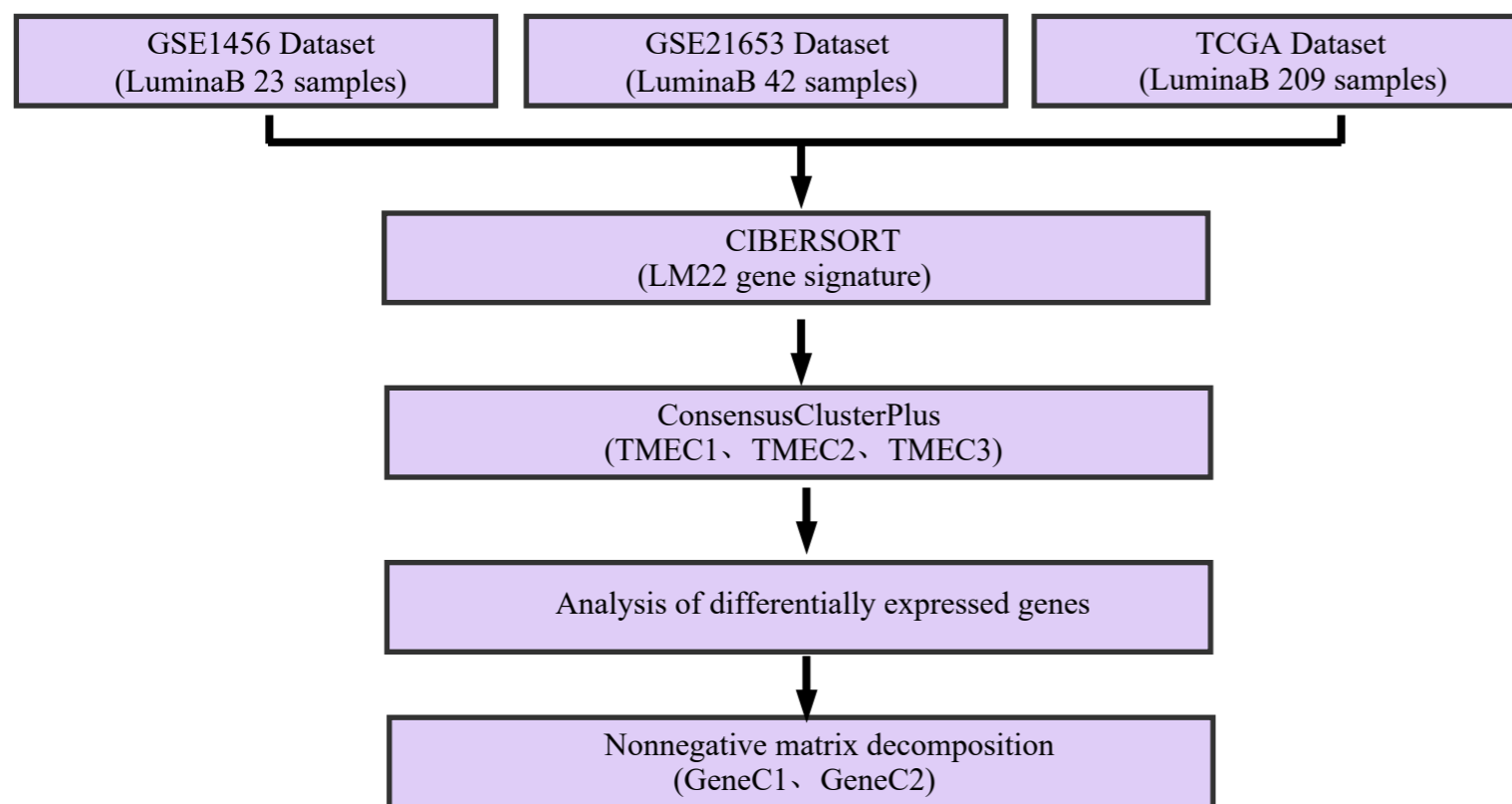

B

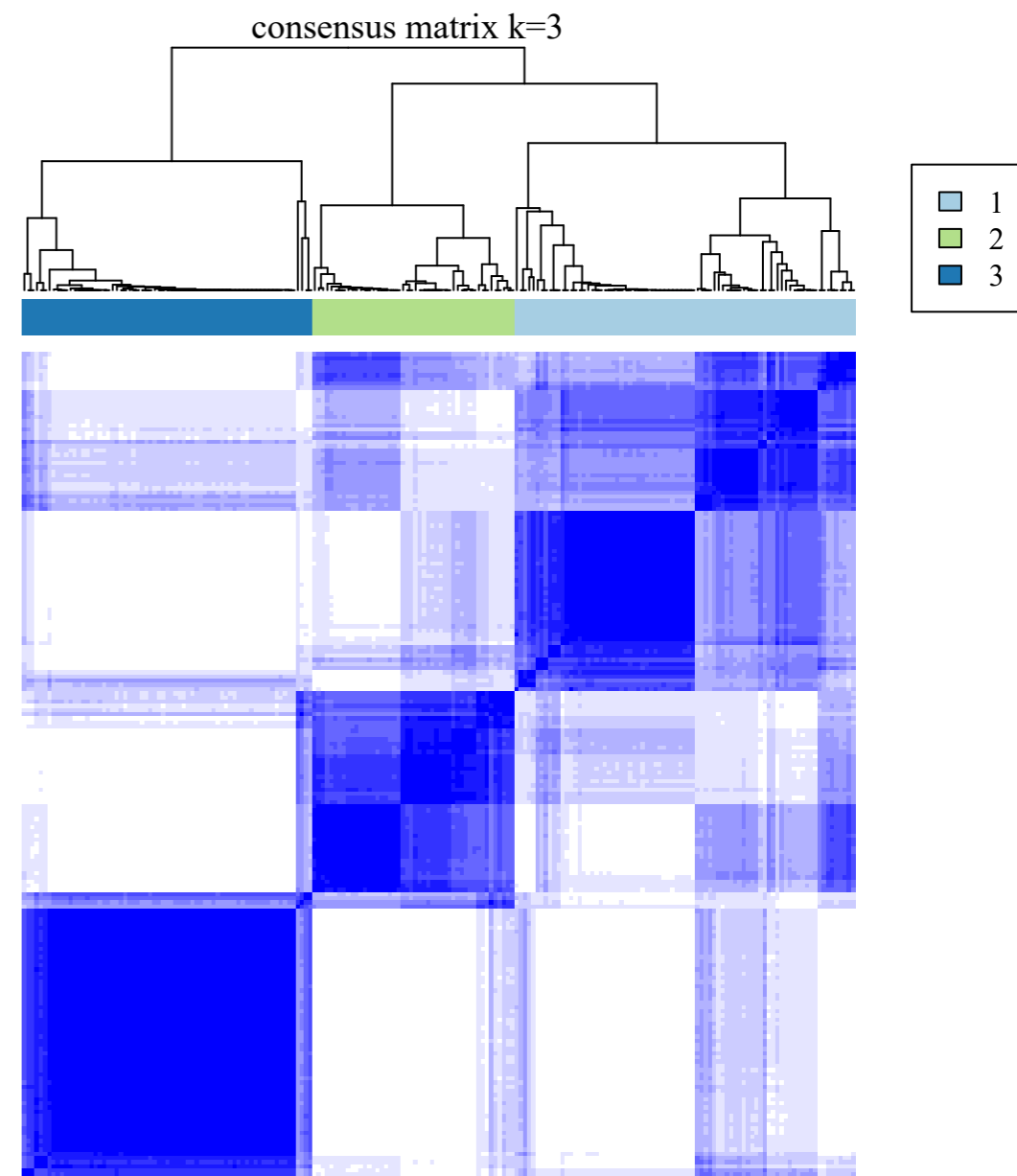

C

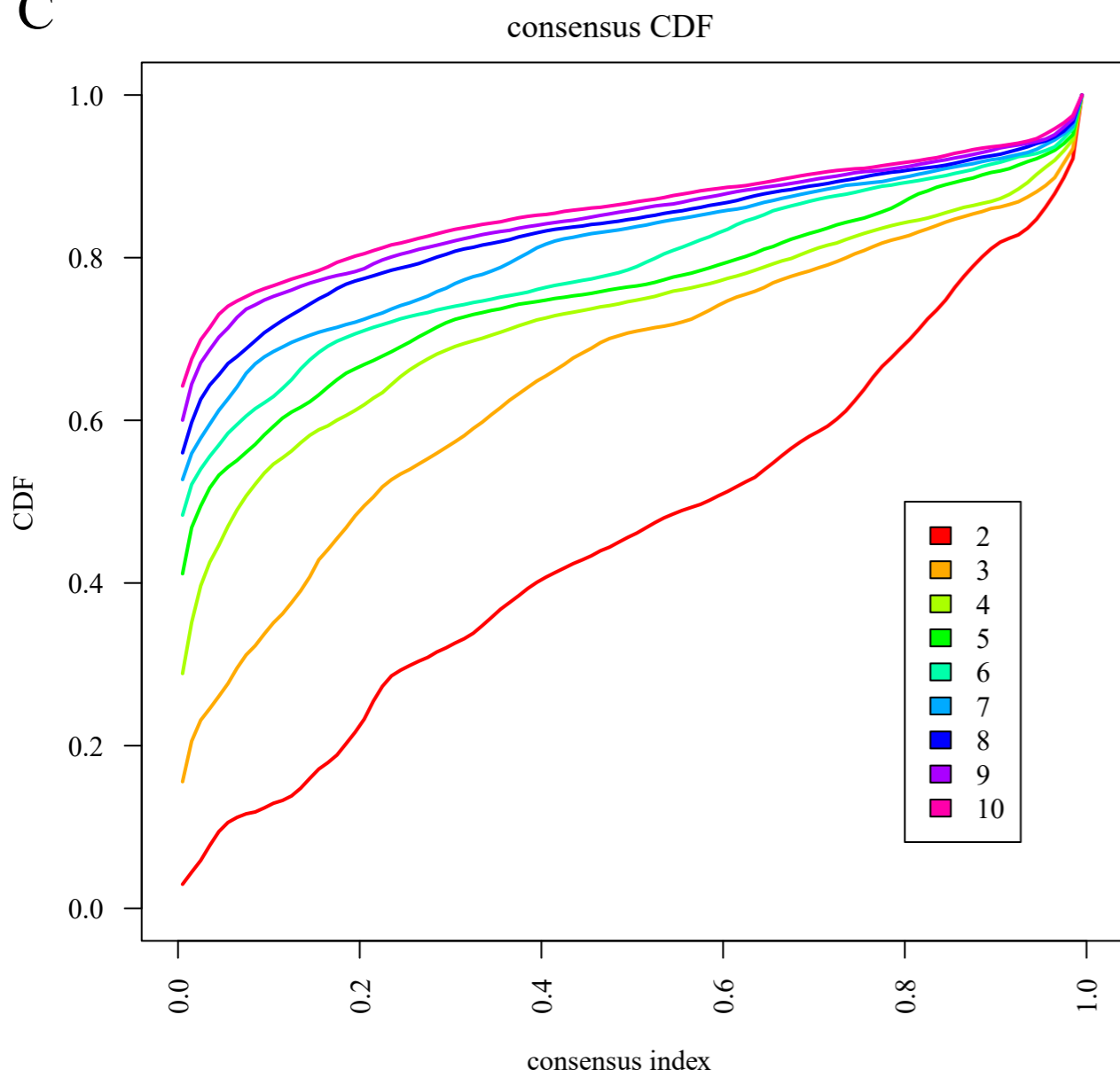

D

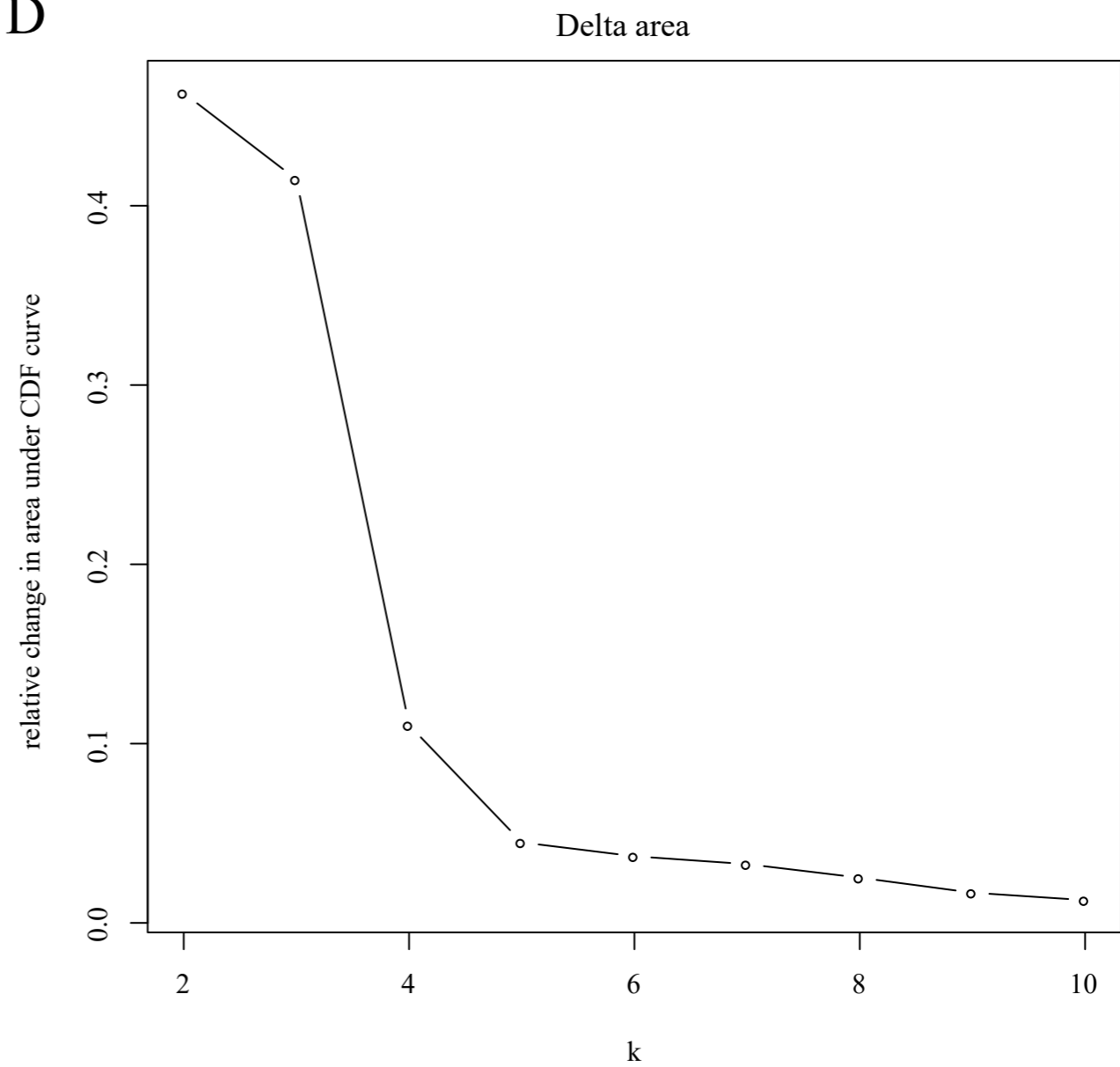

Supplement: Supplementary 1 — Figure S1: A: work flow chart. B: consensus matrix for TME-infiltrating cell classification with the corresponding heat map. The colour-coded heat map corresponding to the consensus matrix for k = 3 obtained by applying consensus clustering. The colour gradients were from 0 to 1, representing the degree of consensus, with white corresponding to 0 and dark blue to 1. C: the cumulative distribution function (CDF) curves in consensus cluster analysis. CDF curves of consensus scores by different subtype numbers (k = 2, 3, 4, 5, 6, 7, 8, 9, 10) were represented. D: delta area curve of consensus clustering, indicating the relative change in area under the cumulative distribution function (CDF) curve for each category number k compared with k–1. The horizontal axis represents the category number k, and the vertical axis represents the relative change in area under CDF curve. [file 5621441.f1.pdf]

A

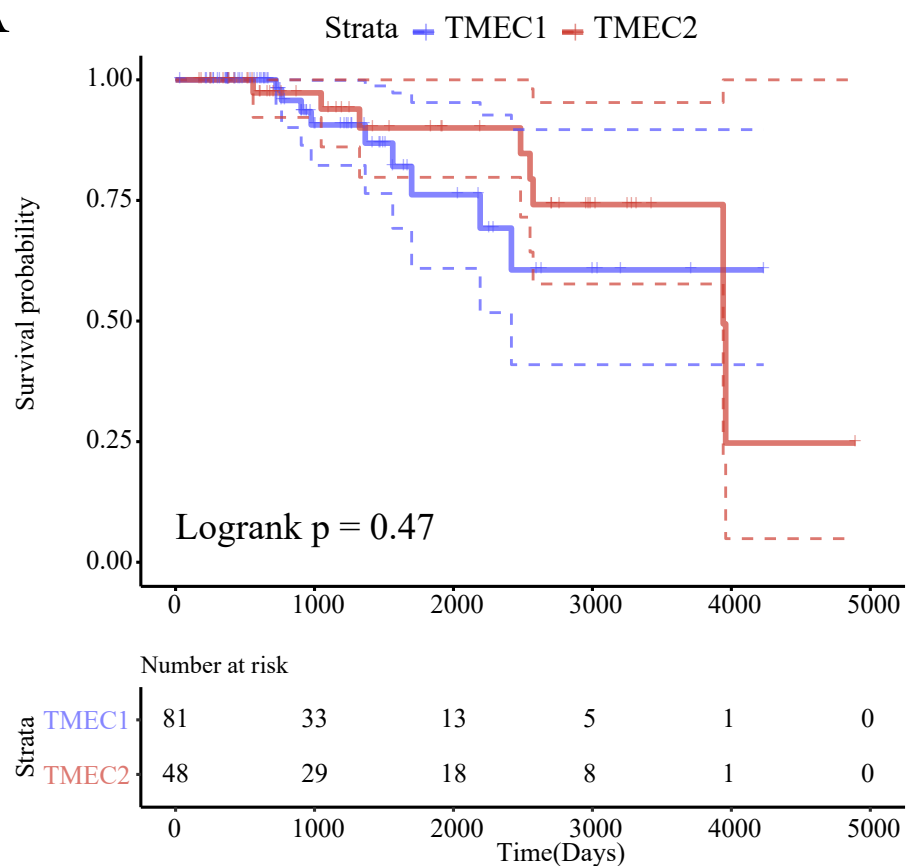

B

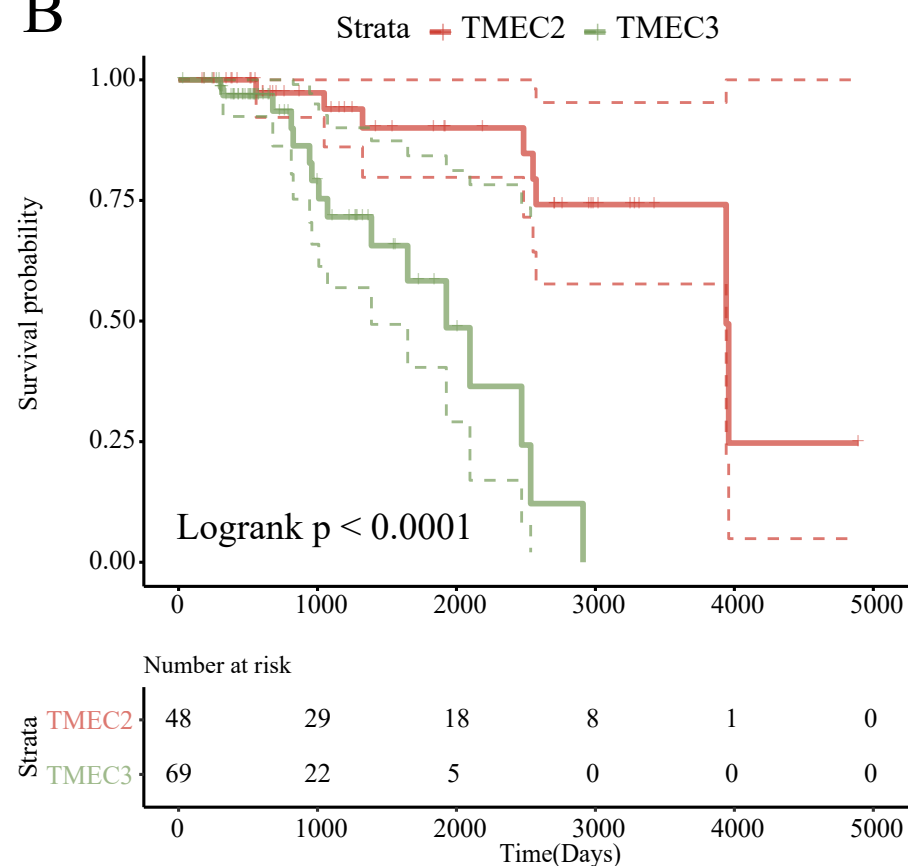

C

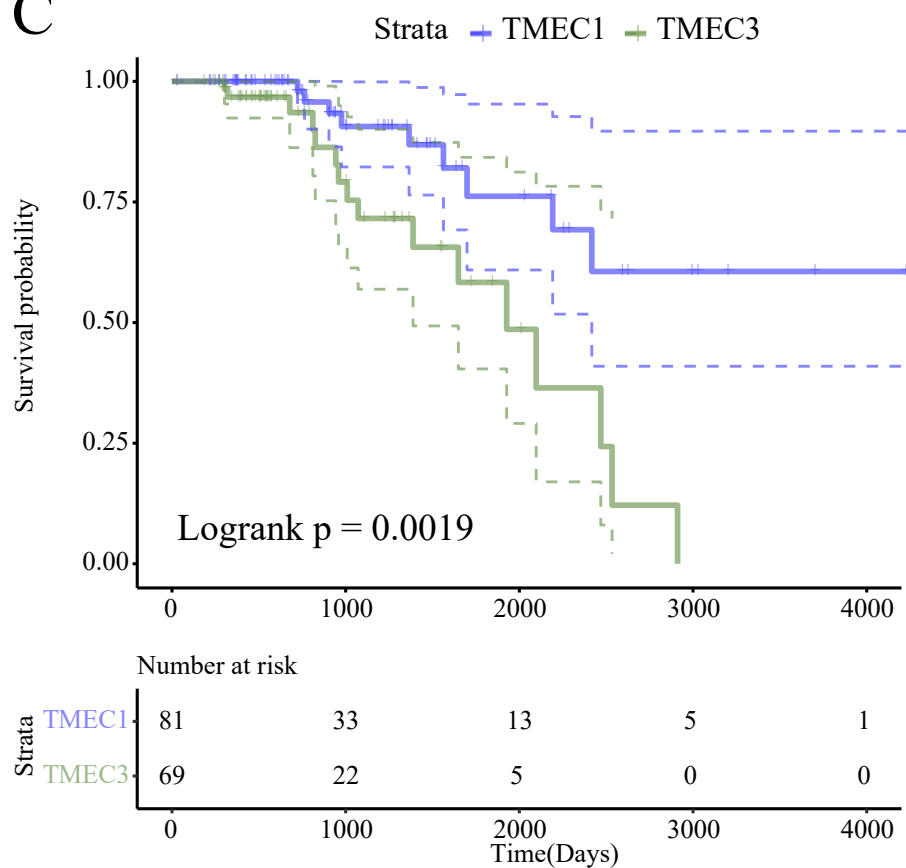

D

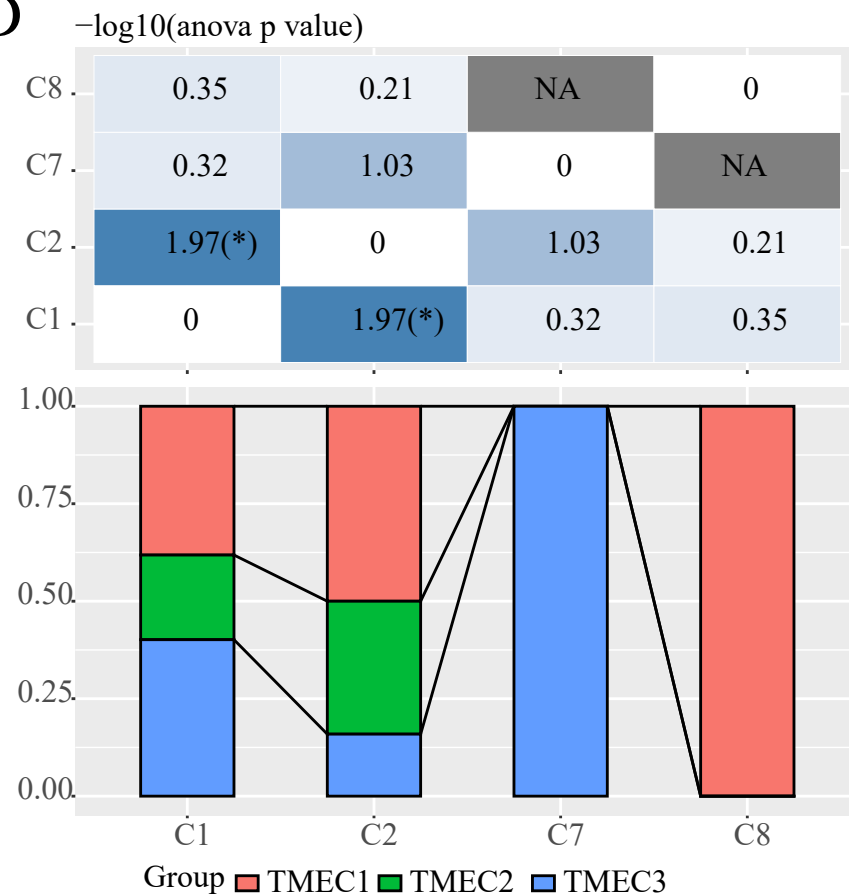

Supplement: Supplementary 2 — Figure S2: A: prognosis KM curve of TMC1 vs. TMEC2. B: prognosis KM curve of TMC2 vs. TMEC3. C: prognosis KM curve of TMC1 vs. TMEC3. D: the molecular subtypes of TCGA samples were compared with the mRNA subtypes of Berger et al. [file 5621441.f2.pdf]

A

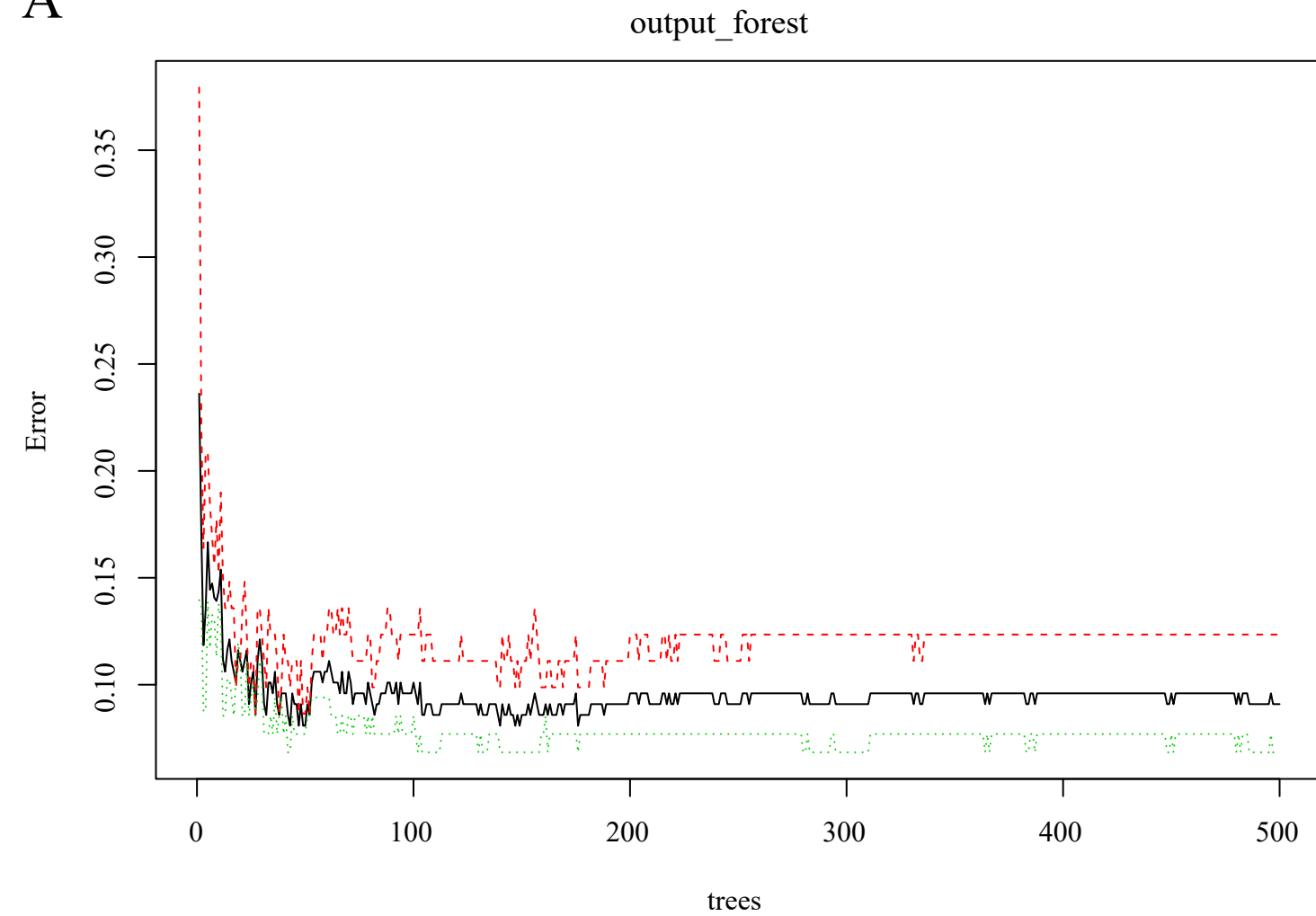

B

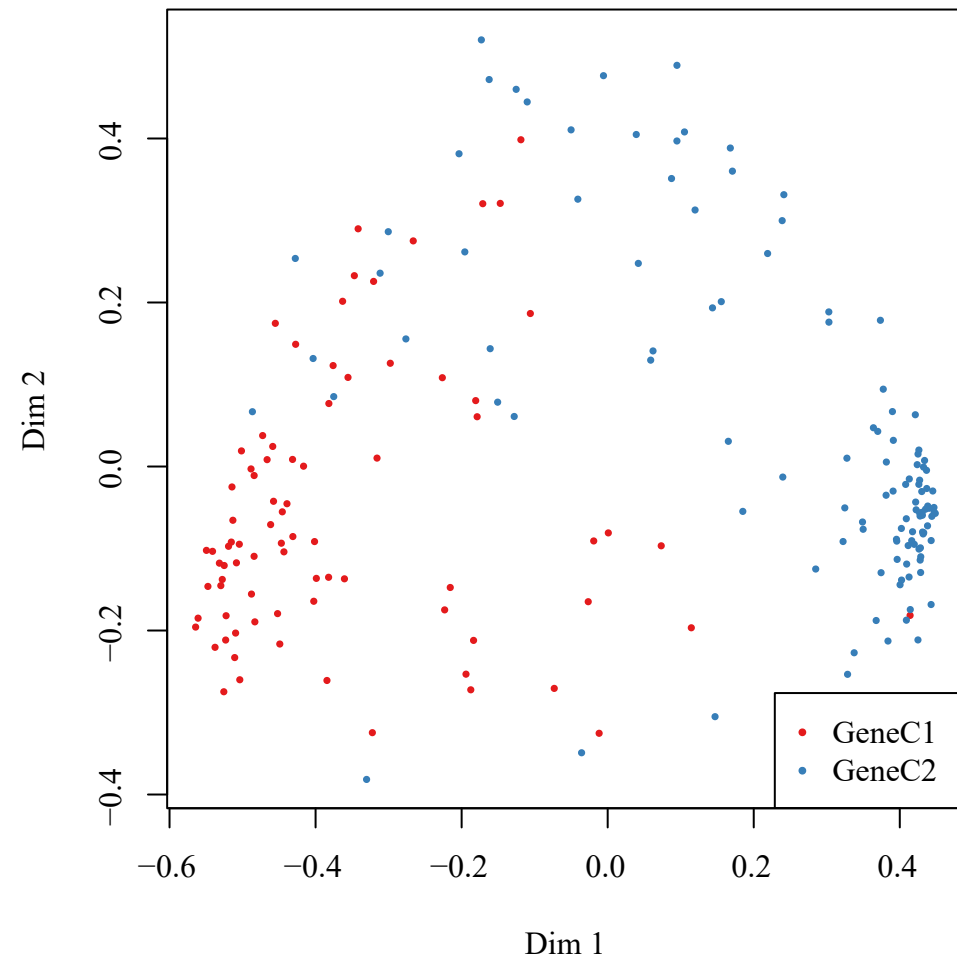

C

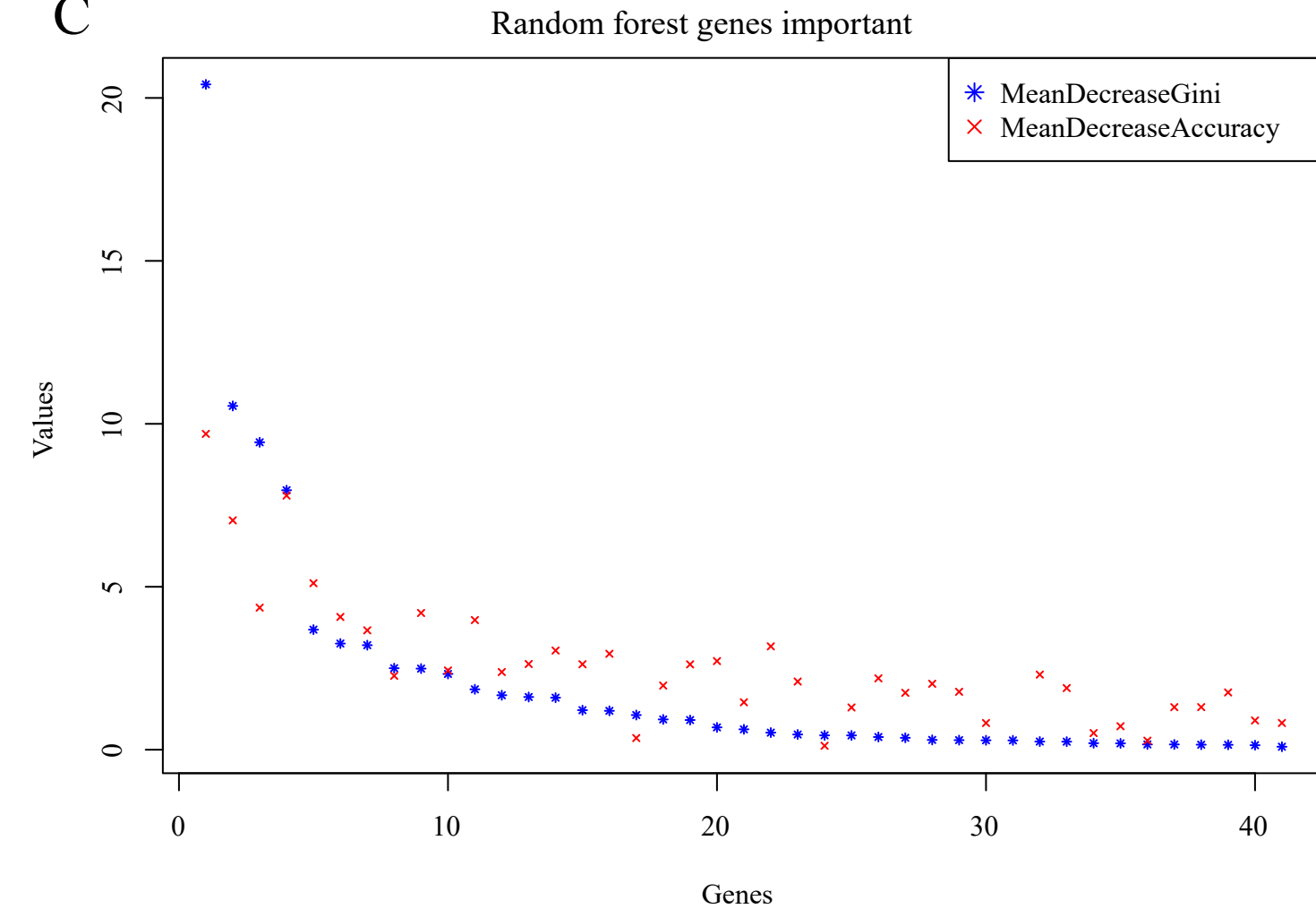

Supplement: Supplementary 3 — Figure S3: A: the distribution of random forest error rate varying with tree parameters. B: multidimensional scaling (MDS) plot for GeneCluster (Gene C1 and Gene C2) data. C: random forest importance for DEGs with mean decrease Gini index (blue) and mean decrease accuracy (red). [file 5621441.f3.pdf]

A

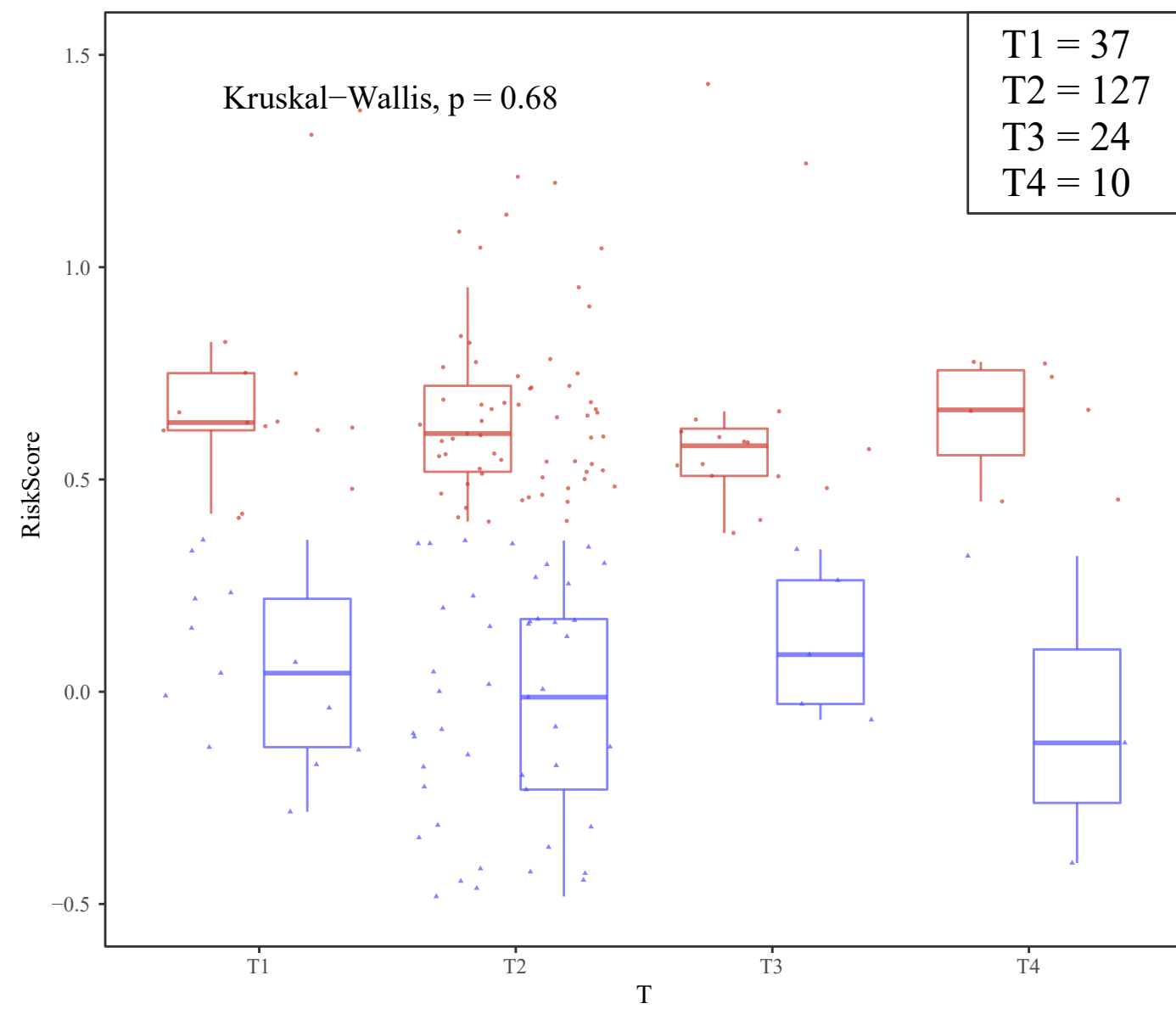

B

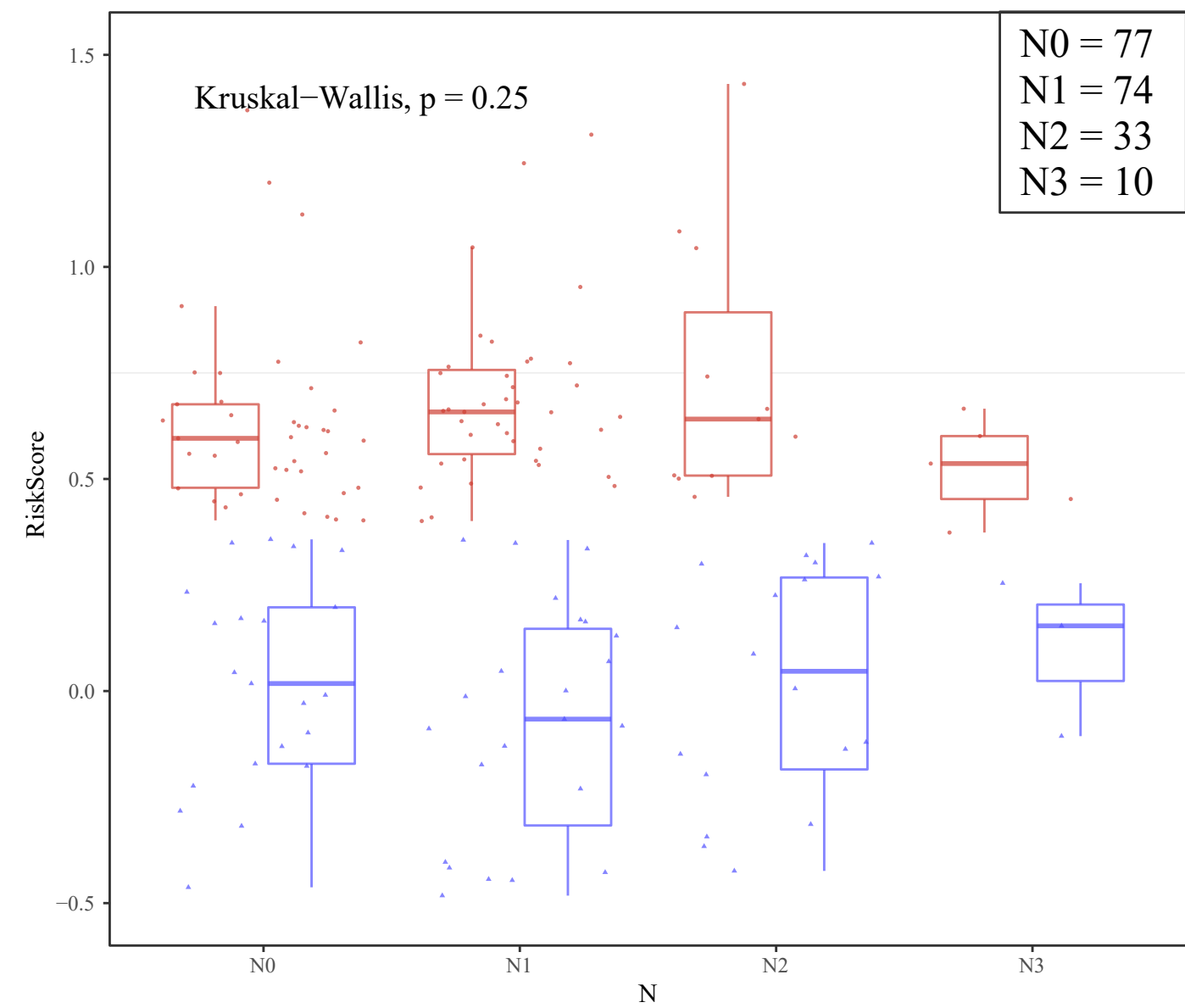

C

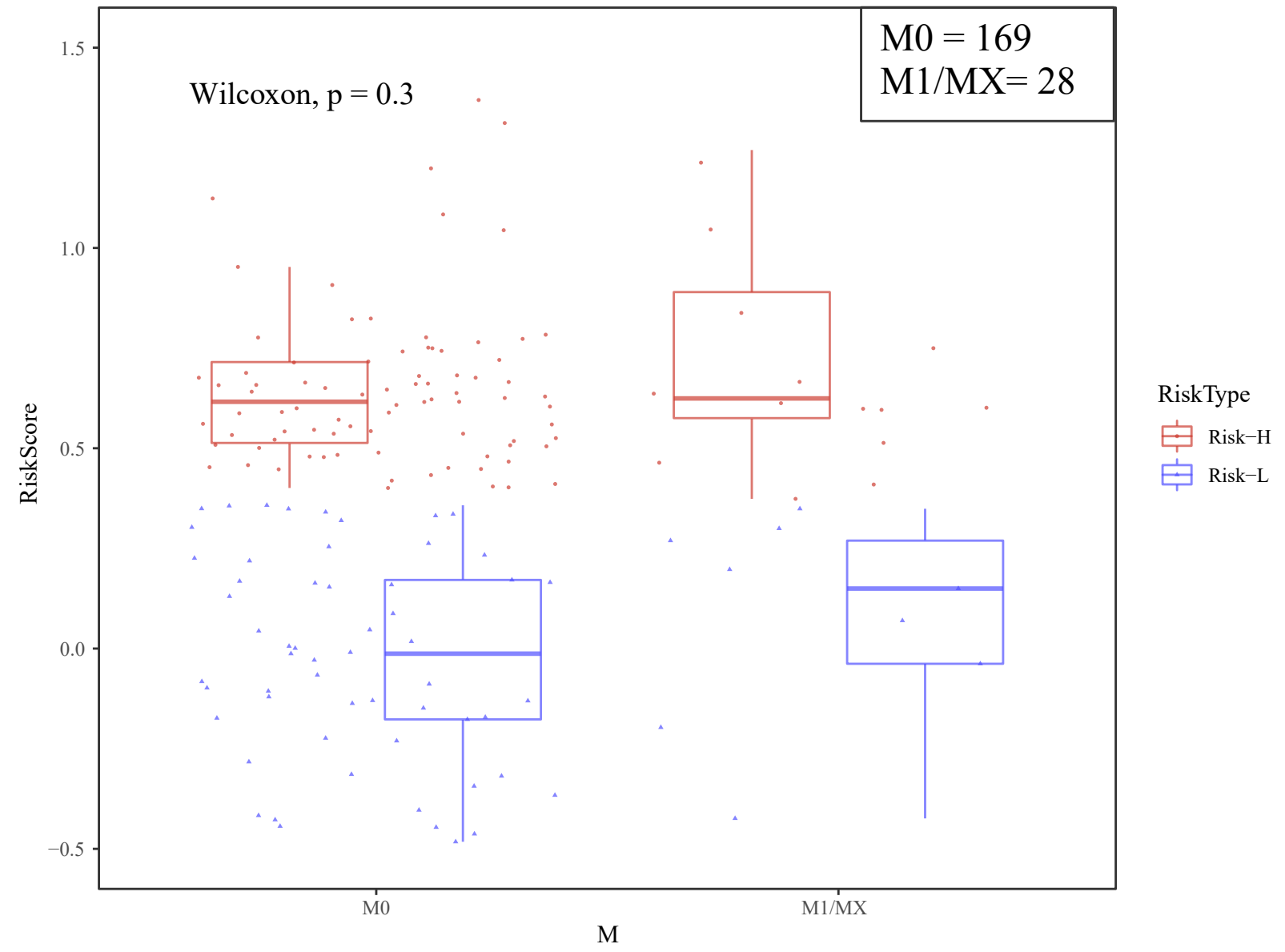

D

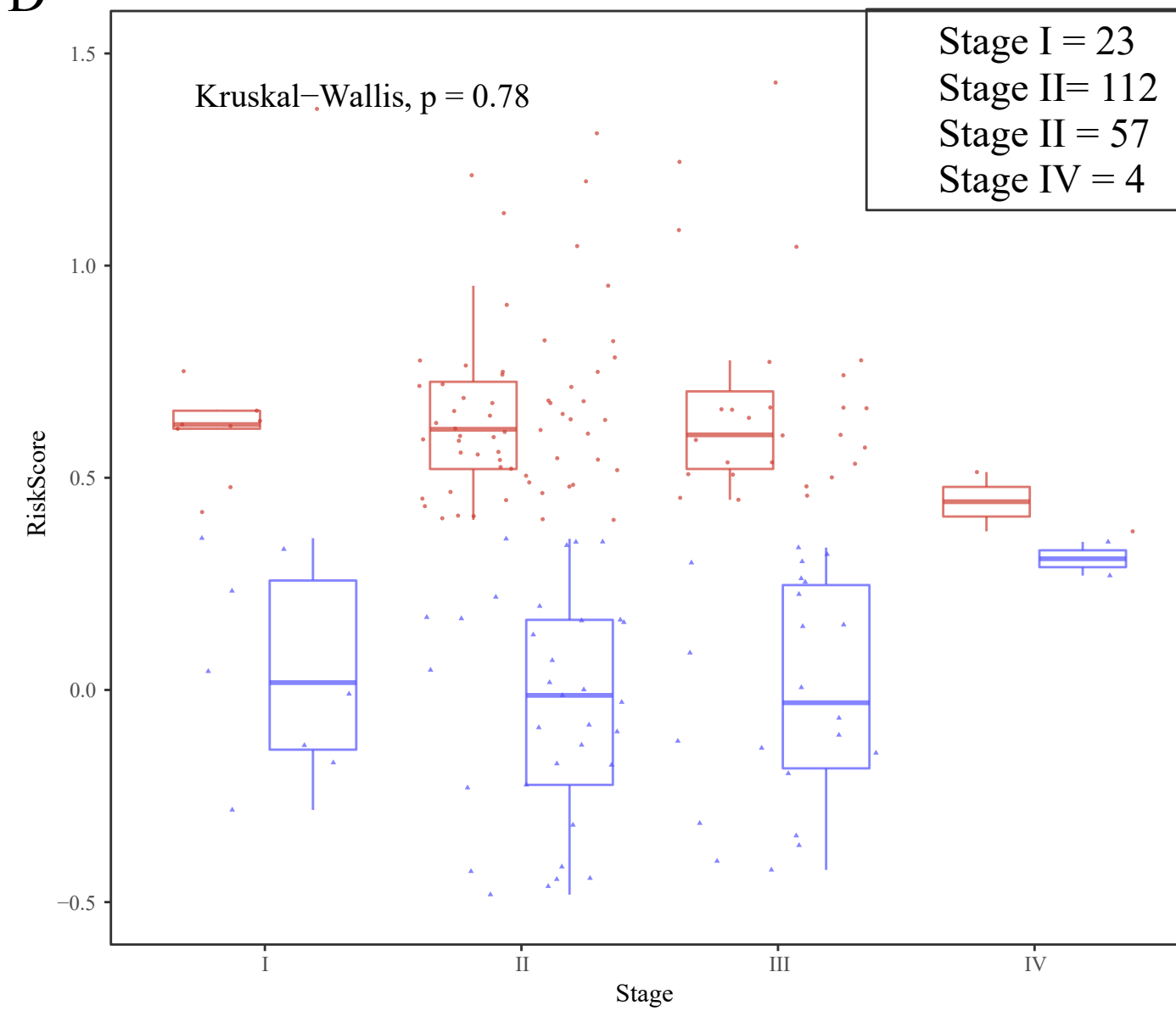

E

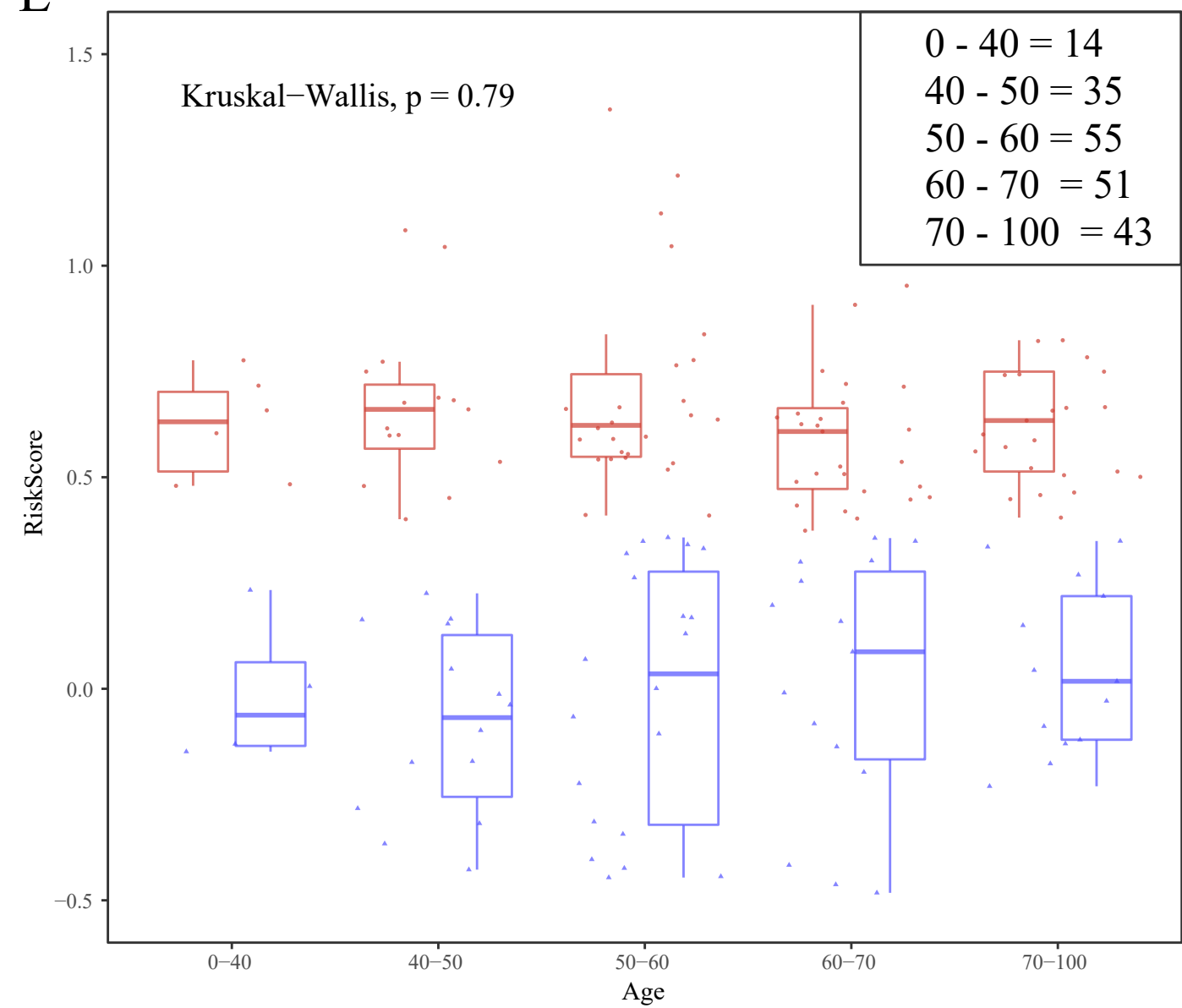

Supplement: Supplementary 4 — Figure S4: the relationship between TME scores and clinical characteristics found no significant difference. A: the relationship between TME scores and T stage. B: the relationship between TME scores and N stage. C: the relationship between TME scores and M stage. D: the relationship between TME scores and stage. E: the relationship between TME scores and age. [file 5621441.f4.pdf]

A

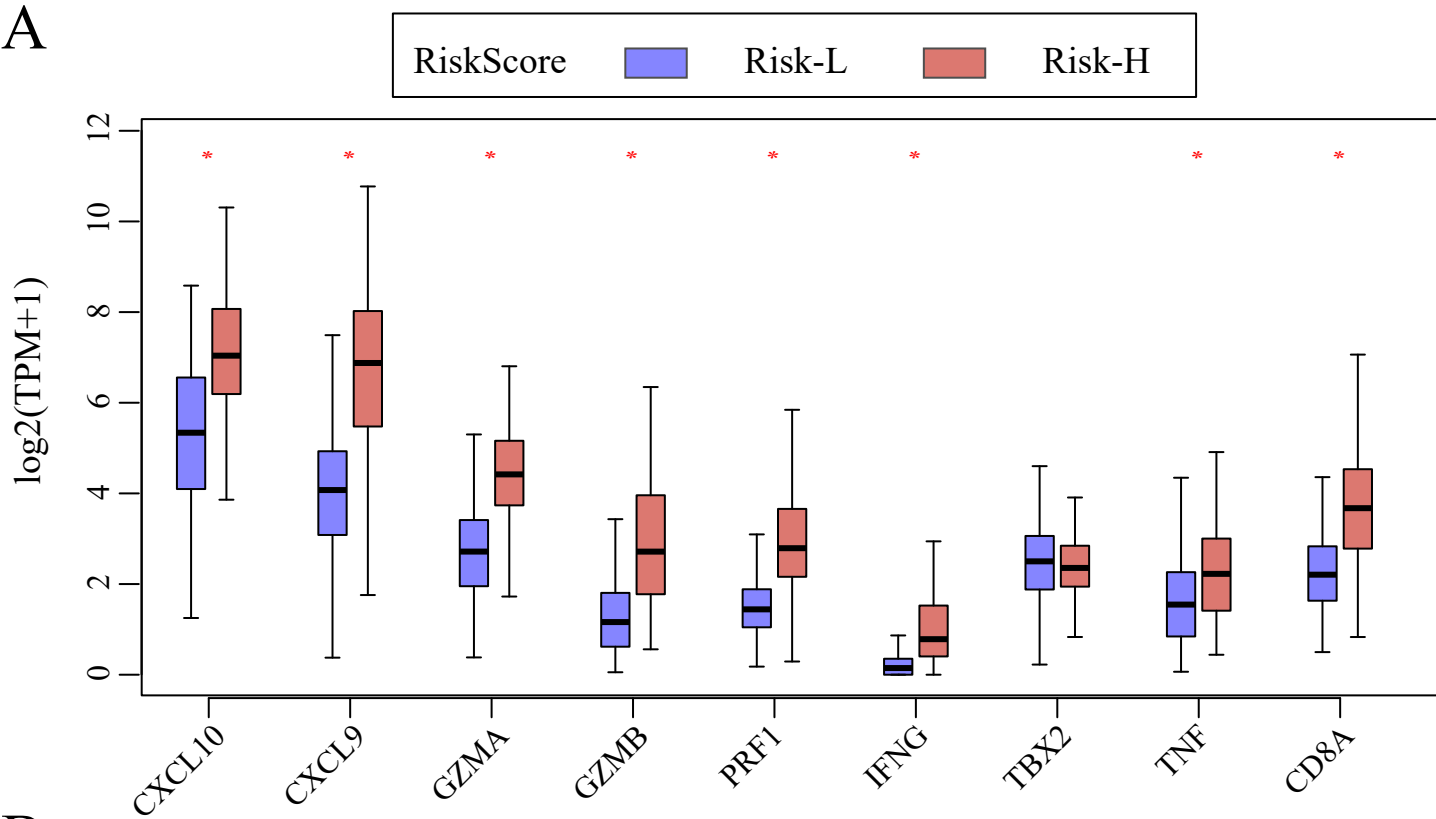

B

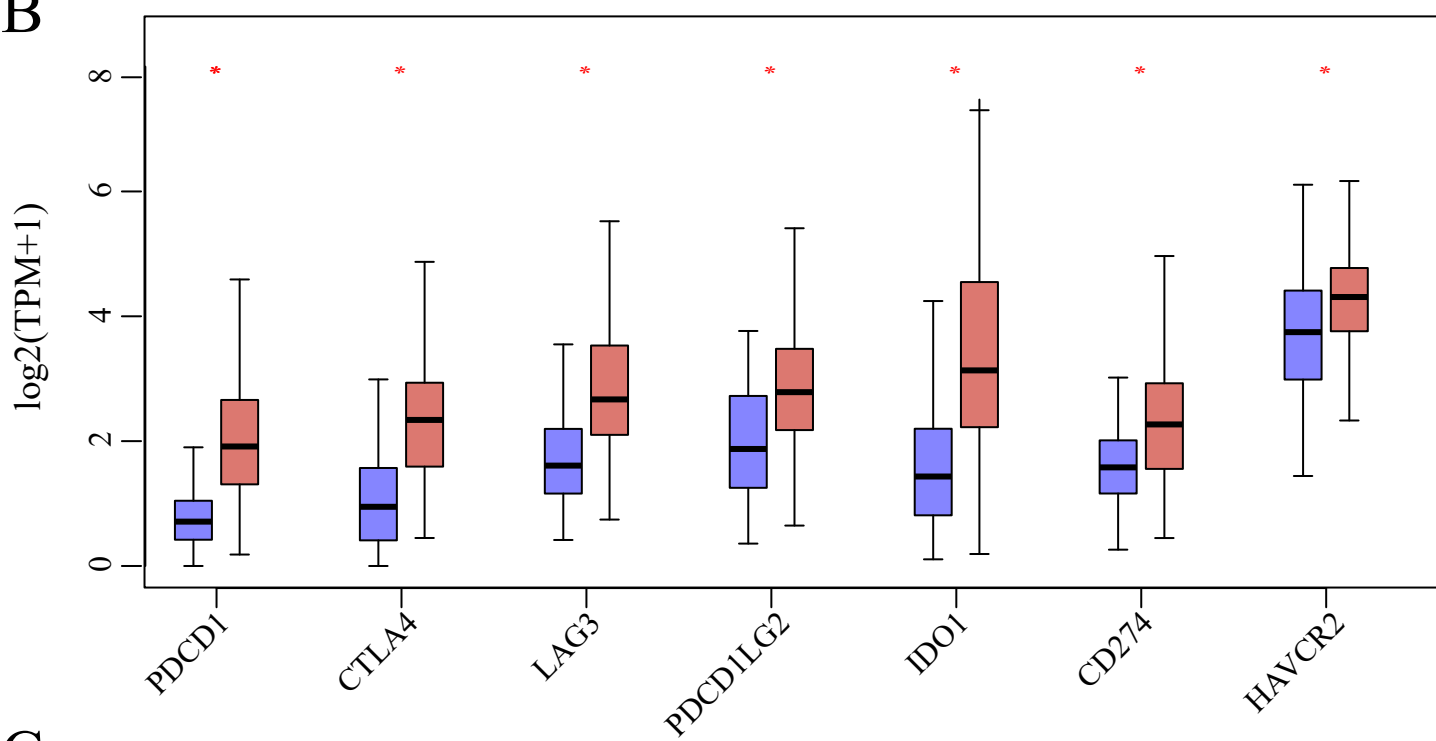

C

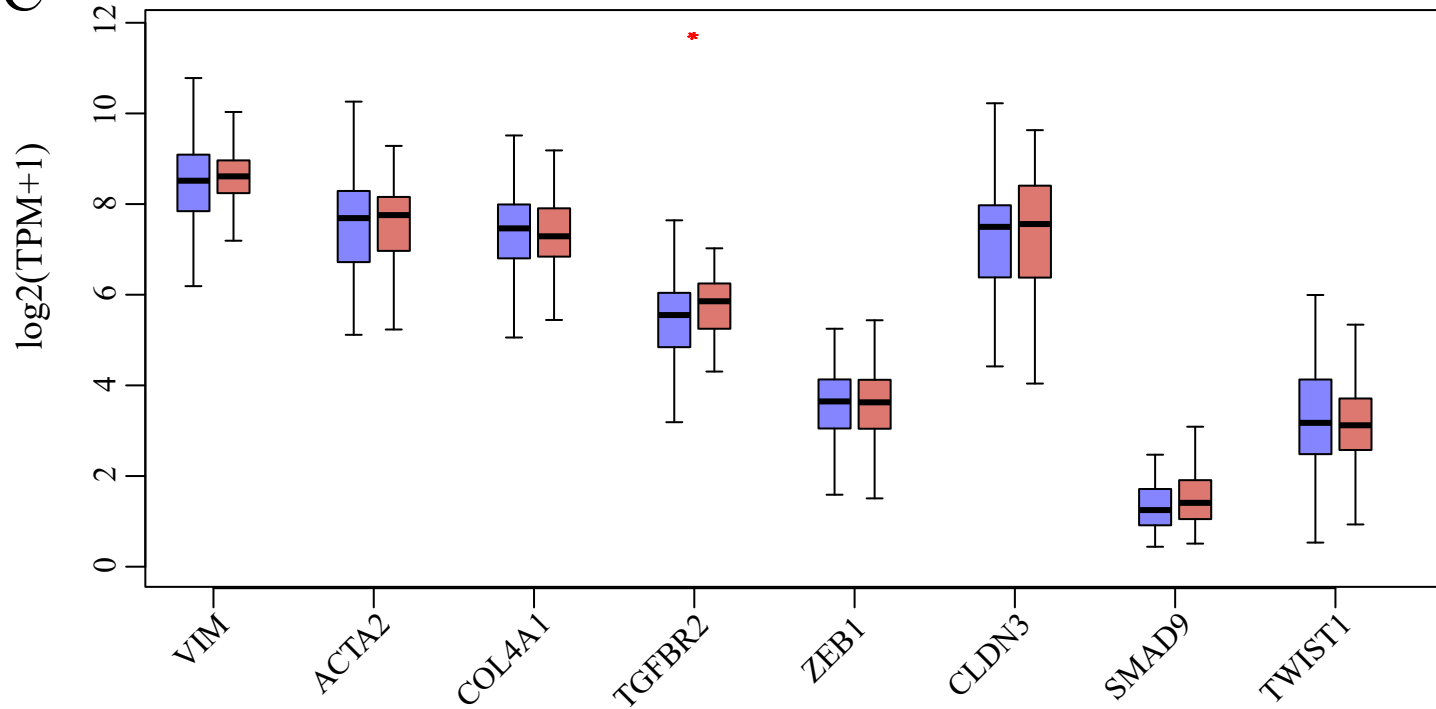

Supplement: Supplementary 5 — Figure S5: A: the expressions of immune activation genes (CXCL10, CXCL9, GZMA, GZMB, PRF1, IFNG, TBX2, TNF, and CD8A) in risk-l and risk-h samples. B: the expressions of immune checkpoint genes (PDCD1, CTLA4, LAG3, IDO1, CD274, PDCD1LG2, and HAVCR2) in risk-l and risk-h samples. C: the expressions of TGF/EMT pathway genes VIM, ACTA2, COL4A1, TGFBR2, ZEB1, CLDN3, SMAD9, and TWIST1 in risk-l and risk-h samples. [file 5621441.f5.pdf]

A

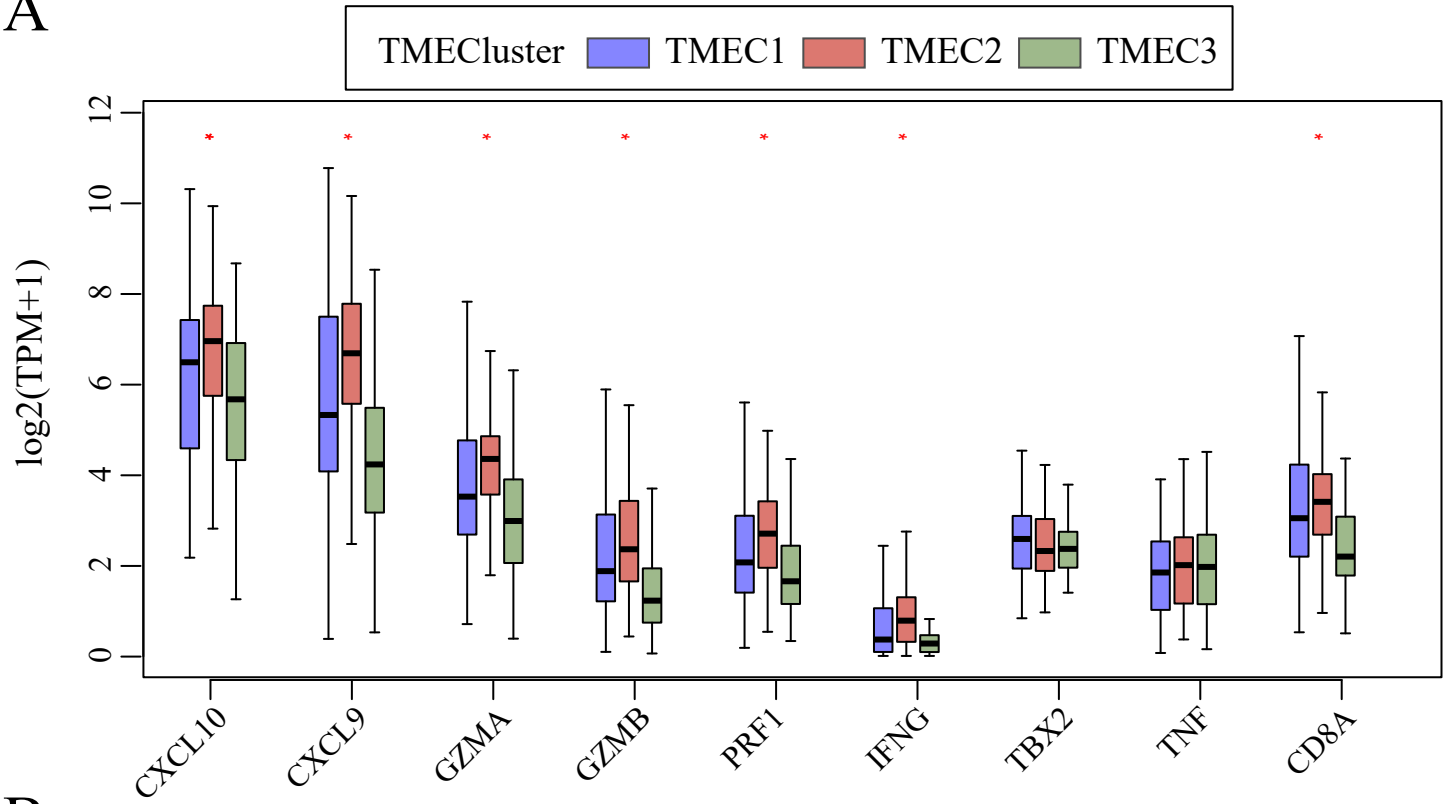

B

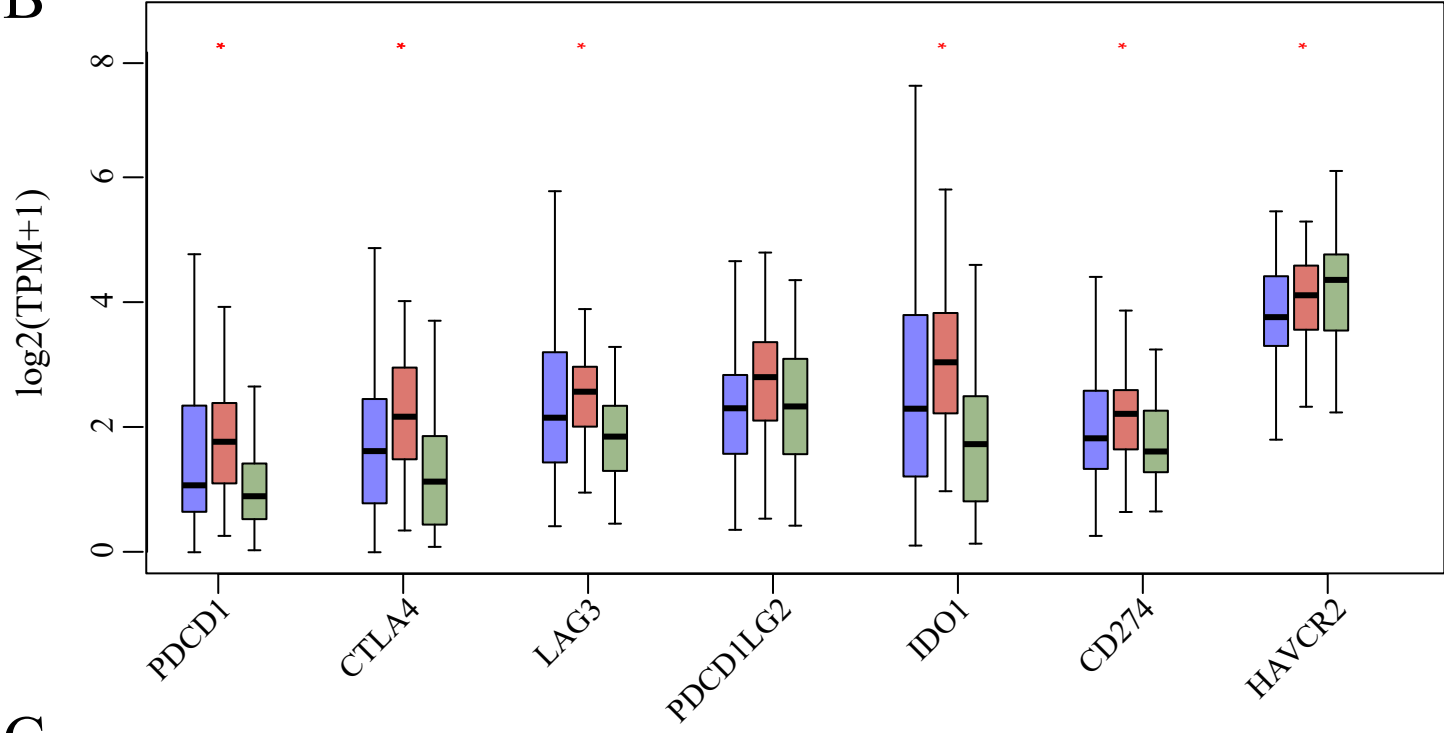

C

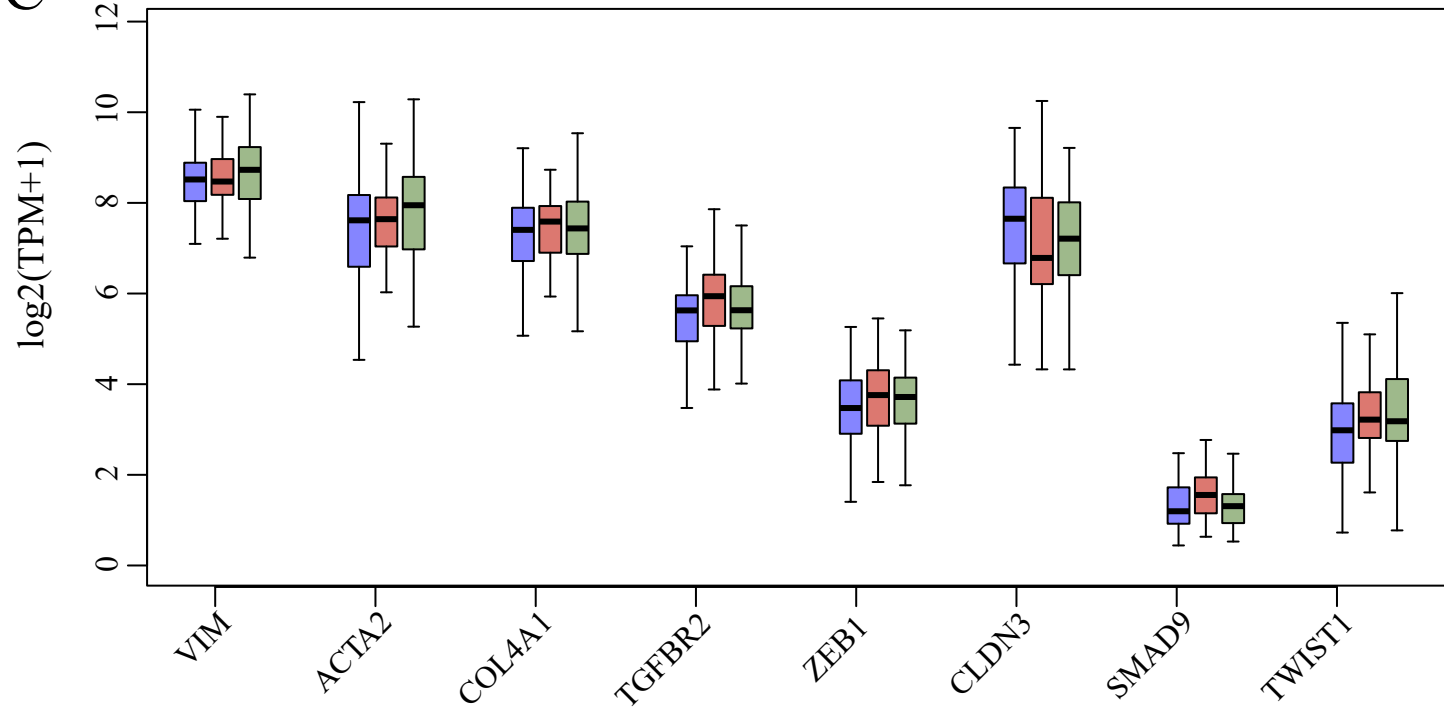

Supplement: Supplementary 6 — Figure S6: A: the expressions of immune activation genes (CXCL10, CXCL9, GZMA, GZMB, PRF1, IFNG, TBX2, TNF, and CD8A) in TMEC. B: the expressions of immune checkpoint genes (PDCD1, CTLA4, LAG3, IDO1, CD274, PDCD1LG2, and HAVCR2) in TMEC. C: the expressions of TGF/EMT pathway genes VIM, ACTA2, COL4A1, TGFBR2, ZEB1, CLDN3, SMAD9, and TWIST1 in TMEC. [file 5621441.f6.pdf]
