# Supplementary material for: Prognosis of Tumor Microenvironment in Luminal B-Type Breast Cancer
Source: Dis Markers. 2022 Feb 10;2022:5621441. doi: 10.1155/2022/5621441 (PMC8886761; doi:10.1155/2022/5621441)
Supplement: Supplementary 7 — Supplementary Table 1: molecular subtypes of TCGA samples. [file 5621441.f7.docx]

Supplementary Table1: Molecular subtypes of TCGA samples

| Sample | SubType |
| --- | --- |
| TCGA-A8-A09N | TMEC1 |
| TCGA-E9-A249 | TMEC1 |
| TCGA-AN-A0AK | TMEC1 |
| TCGA-AC-A7VB | TMEC1 |
| TCGA-A8-A09R | TMEC1 |
| TCGA-E2-A15K | TMEC1 |
| TCGA-5T-A9QA | TMEC1 |
| TCGA-AN-A0AJ | TMEC1 |
| TCGA-C8-A1HL | TMEC1 |
| TCGA-C8-A12M | TMEC1 |
| TCGA-A8-A08I | TMEC1 |
| TCGA-C8-A130 | TMEC1 |
| TCGA-D8-A27W | TMEC1 |
| TCGA-C8-A1HM | TMEC1 |
| TCGA-LL-A7T0 | TMEC1 |
| TCGA-C8-A26W | TMEC1 |
| TCGA-C8-A12U | TMEC1 |
| TCGA-S3-AA17 | TMEC1 |
| TCGA-D8-A1J8 | TMEC1 |
| TCGA-A8-A084 | TMEC1 |
| TCGA-D8-A1XF | TMEC1 |
| TCGA-D8-A1JC | TMEC1 |
| TCGA-E9-A2JS | TMEC1 |
| TCGA-E2-A9RU | TMEC1 |
| TCGA-AR-A0TY | TMEC1 |
| TCGA-S3-A6ZF | TMEC1 |
| TCGA-A2-A1G4 | TMEC1 |
| TCGA-A8-A08G | TMEC1 |
| TCGA-AO-A0J7 | TMEC1 |
| TCGA-A1-A0SI | TMEC1 |
| TCGA-E2-A155 | TMEC1 |
| TCGA-E9-A1RG | TMEC1 |
| TCGA-A2-A0SW | TMEC1 |
| TCGA-A2-A0YG | TMEC1 |
| TCGA-EW-A2FW | TMEC1 |
| TCGA-A2-A0YT | TMEC1 |
| TCGA-E9-A54Y | TMEC1 |
| TCGA-E9-A54X | TMEC1 |
| TCGA-C8-A27A | TMEC1 |
| TCGA-AQ-A04H | TMEC1 |
| TCGA-BH-A1F8 | TMEC1 |
| TCGA-A2-A0D4 | TMEC1 |
| TCGA-E9-A5UO | TMEC1 |
| TCGA-EW-A1OY | TMEC1 |
| TCGA-EW-A1OX | TMEC1 |
| TCGA-A7-A0CJ | TMEC1 |
| TCGA-A8-A092 | TMEC1 |
| TCGA-A8-A08P | TMEC1 |
| TCGA-A8-A07L | TMEC1 |
| TCGA-E9-A1RB | TMEC1 |
| TCGA-A8-A08S | TMEC1 |
| TCGA-A8-A09M | TMEC1 |
| TCGA-E9-A22A | TMEC1 |
| TCGA-E2-A10C | TMEC1 |
| TCGA-E9-A22H | TMEC1 |
| TCGA-E9-A22D | TMEC1 |
| TCGA-E9-A22E | TMEC1 |
| TCGA-BH-A0C0 | TMEC1 |
| TCGA-E2-A14O | TMEC1 |
| TCGA-E2-A109 | TMEC1 |
| TCGA-E9-A1R7 | TMEC1 |
| TCGA-3C-AALJ | TMEC1 |
| TCGA-A8-A09E | TMEC1 |
| TCGA-A2-A0T3 | TMEC1 |
| TCGA-BH-A18U | TMEC1 |
| TCGA-E2-A15T | TMEC1 |
| TCGA-A8-A076 | TMEC1 |
| TCGA-AO-A03N | TMEC1 |
| TCGA-A2-A0CY | TMEC1 |
| TCGA-AO-A0JM | TMEC1 |
| TCGA-BH-A1FN | TMEC1 |
| TCGA-BH-A0BZ | TMEC1 |
| TCGA-AR-A0TV | TMEC1 |
| TCGA-B6-A0WV | TMEC1 |
| TCGA-GM-A2DO | TMEC1 |
| TCGA-AR-A1AW | TMEC1 |
| TCGA-A2-A25E | TMEC1 |
| TCGA-AR-A24Z | TMEC1 |
| TCGA-AR-A24N | TMEC1 |
| TCGA-A2-A04R | TMEC1 |
| TCGA-B6-A1KN | TMEC1 |
| TCGA-AN-A0FF | TMEC2 |
| TCGA-E9-A1R4 | TMEC2 |
| TCGA-E2-A56Z | TMEC2 |
| TCGA-EW-A1IY | TMEC2 |
| TCGA-AO-A1KS | TMEC2 |
| TCGA-C8-A8HQ | TMEC2 |
| TCGA-BH-A0HU | TMEC2 |
| TCGA-E2-A15S | TMEC2 |
| TCGA-B6-A0WW | TMEC2 |
| TCGA-A2-A25C | TMEC2 |
| TCGA-A2-A1FW | TMEC2 |
| TCGA-BH-A0BD | TMEC2 |
| TCGA-D8-A1XL | TMEC2 |
| TCGA-C8-A26V | TMEC2 |
| TCGA-A2-A4S3 | TMEC2 |
| TCGA-A7-A2KD | TMEC2 |
| TCGA-E2-A15A | TMEC2 |
| TCGA-A8-A09Q | TMEC2 |
| TCGA-E9-A226 | TMEC2 |
| TCGA-EW-A1J6 | TMEC2 |
| TCGA-EW-A1P0 | TMEC2 |
| TCGA-A2-A04Y | TMEC2 |
| TCGA-E9-A3HO | TMEC2 |
| TCGA-A1-A0SN | TMEC2 |
| TCGA-BH-A0BF | TMEC2 |
| TCGA-B6-A0IN | TMEC2 |
| TCGA-E9-A1RE | TMEC2 |
| TCGA-BH-A0HY | TMEC2 |
| TCGA-AR-A0U2 | TMEC2 |
| TCGA-E2-A1L7 | TMEC2 |
| TCGA-BH-A0AU | TMEC2 |
| TCGA-A2-A0EY | TMEC2 |
| TCGA-AO-A0JD | TMEC2 |
| TCGA-AO-A03O | TMEC2 |
| TCGA-BH-A0C3 | TMEC2 |
| TCGA-AR-A250 | TMEC2 |
| TCGA-B6-A0IB | TMEC2 |
| TCGA-BH-A0C7 | TMEC2 |
| TCGA-AO-A1KP | TMEC2 |
| TCGA-AR-A24S | TMEC2 |
| TCGA-AR-A0TQ | TMEC2 |
| TCGA-AC-A2BM | TMEC2 |
| TCGA-Z7-A8R6 | TMEC2 |
| TCGA-A2-A0CW | TMEC2 |
| TCGA-AR-A0TT | TMEC2 |
| TCGA-AR-A24R | TMEC2 |
| TCGA-BH-A209 | TMEC2 |
| TCGA-AR-A24H | TMEC2 |
| TCGA-A8-A06Q | TMEC3 |
| TCGA-A8-A06Z | TMEC3 |
| TCGA-E9-A1NF | TMEC3 |
| TCGA-EW-A1PC | TMEC3 |
| TCGA-A8-A07S | TMEC3 |
| TCGA-LL-A5YM | TMEC3 |
| TCGA-A8-A079 | TMEC3 |
| TCGA-E9-A1NI | TMEC3 |
| TCGA-D8-A1Y1 | TMEC3 |
| TCGA-A8-A07W | TMEC3 |
| TCGA-D8-A27R | TMEC3 |
| TCGA-A7-A6VX | TMEC3 |
| TCGA-BH-A42T | TMEC3 |
| TCGA-C8-A1HG | TMEC3 |
| TCGA-C8-A12W | TMEC3 |
| TCGA-C8-A1HN | TMEC3 |
| TCGA-C8-A3M8 | TMEC3 |
| TCGA-A8-A06O | TMEC3 |
| TCGA-D8-A1JT | TMEC3 |
| TCGA-D8-A1Y3 | TMEC3 |
| TCGA-D8-A1Y2 | TMEC3 |
| TCGA-E9-A1N6 | TMEC3 |
| TCGA-BH-A0H0 | TMEC3 |
| TCGA-D8-A1XZ | TMEC3 |
| TCGA-D8-A1XR | TMEC3 |
| TCGA-C8-A274 | TMEC3 |
| TCGA-D8-A1X7 | TMEC3 |
| TCGA-A8-A075 | TMEC3 |
| TCGA-D8-A27N | TMEC3 |
| TCGA-D8-A1J9 | TMEC3 |
| TCGA-AO-A1KT | TMEC3 |
| TCGA-D8-A1X6 | TMEC3 |
| TCGA-A8-A06R | TMEC3 |
| TCGA-A8-A082 | TMEC3 |
| TCGA-D8-A1JD | TMEC3 |
| TCGA-D8-A1X5 | TMEC3 |
| TCGA-D8-A1JE | TMEC3 |
| TCGA-D8-A1JI | TMEC3 |
| TCGA-D8-A1JJ | TMEC3 |
| TCGA-AO-A0J3 | TMEC3 |
| TCGA-A2-A0YH | TMEC3 |
| TCGA-BH-A0W3 | TMEC3 |
| TCGA-A7-A13F | TMEC3 |
| TCGA-BH-A202 | TMEC3 |
| TCGA-BH-A18L | TMEC3 |
| TCGA-A2-A0SV | TMEC3 |
| TCGA-A8-A06X | TMEC3 |
| TCGA-BH-A1F2 | TMEC3 |
| TCGA-A8-A08F | TMEC3 |
| TCGA-BH-A1FD | TMEC3 |
| TCGA-E9-A1NA | TMEC3 |
| TCGA-EW-A1OZ | TMEC3 |
| TCGA-A8-A095 | TMEC3 |
| TCGA-E9-A228 | TMEC3 |
| TCGA-A2-A25B | TMEC3 |
| TCGA-AR-A2LK | TMEC3 |
| TCGA-B6-A1KC | TMEC3 |
| TCGA-B6-A0X5 | TMEC3 |
| TCGA-A8-A09I | TMEC3 |
| TCGA-BH-A1FM | TMEC3 |
| TCGA-AR-A24K | TMEC3 |
| TCGA-BH-A0HW | TMEC3 |
| TCGA-D8-A13Y | TMEC3 |
| TCGA-A2-A1FX | TMEC3 |
| TCGA-AO-A03P | TMEC3 |
| TCGA-BH-A1FJ | TMEC3 |
| TCGA-AR-A2LL | TMEC3 |
| TCGA-B6-A0RL | TMEC3 |
| TCGA-BH-A204 | TMEC3 |
